# Pursuit tracks chase: exploring the role of eye movements in the detection of chasing

**DOI:** 10.7717/peerj.1243

**Published:** 2015-09-15

**Authors:** Matúš Šimkovic, Birgit Träuble

**Affiliations:** Universität zu Köln, Köln, Germany

**Keywords:** Goal-directed motion, Chasing, Perception of animacy, Eye movements, Visual saliency, Template images, Support vector machines, Classification images, Principal component analysis

## Abstract

We explore the role of eye movements in a chase detection task. Unlike the previous studies, which focused on overall performance as indicated by response speed and chase detection accuracy, we decompose the search process into gaze events such as smooth eye movements and use a data-driven approach to separately describe these gaze events. We measured eye movements of four human subjects engaged in a chase detection task displayed on a computer screen. The subjects were asked to detect two chasing rings among twelve other randomly moving rings. Using principal component analysis and support vector machines, we looked at the template and classification images that describe various stages of the detection process. We showed that the subjects mostly search for pairs of rings that move one after another in the same direction with a distance of 3.5–3.8 degrees. To find such pairs, the subjects first looked for regions with a high ring density and then pursued the rings in this region. Most of these groups consisted of two rings. Three subjects preferred to pursue the pair as a single object, while the remaining subject pursued the group by alternating the gaze between the two individual rings. In the discussion, we argue that subjects do not compare the movement of the pursued pair to a singular preformed template that describes a chasing motion. Rather, subjects bring certain hypotheses about what motion may qualify as chase and then, through feedback, they learn to look for a motion pattern that maximizes their performance.

## Introduction

Imagine aliens landed on Earth yesterday. They deposited a device on the ground and left. The device is the size of a shoebox and has a hard, impenetrable metal shell. It has a camera on one side and a lamp on the opposite side. The lamp flashes with a varying intensity. Scientists were gathered to inspect the strange device. They soon discovered that the device sends a strong light flash when the camera detects motion. After brief experimentation, the scientists found that the device gives an especially strong response to displays of synchronized motion of a pair of blobs. Some researchers went on to design displays that create the strongest response. Others wanted to know what the visual primitives are that influence the device’s response. Is the response stronger when the motion is fast or does the smoothness of the motion matter? Is the response stronger when the moving blobs are near or farther apart? Is the response stronger when the blobs move in the same direction or when they move apart? Other researchers argued that these ways of studying the device are unsystematic and potentially biased because when designing the stimuli for discovering the strongest response or when selecting the primitives that should be probed, researchers rely on their intuitions or inefficient random trial-and-error experimentation at best. Still other researchers argued that the magnitude of response varies with the device’s previous response history. When subjected to weak displays for long periods, the device provided positive response even though under normal conditions it would not do so. Moreover, some researchers pointed out that on the input side there are also additional factors that should be taken into account: with special technology, one may determine what parts of the outside world are being focused by the camera. Finally, some researchers argued that in order to figure out how the device works, one should first find out what its purpose is, based on our knowledge of the alien species.

Reverse-engineering has been repeatedly used as a research metaphor for cognitive science (Dennett, 1994; Tenenbaum et al., 2011). The alien story is specifically tailored to fit the current situation in the research of human perception of animacy and goal-directed motion. Researchers demonstrated that motion provides a sufficient cue for people to ascribe social content to otherwise featureless geometrical objects (Heider & Simmel, 1944). Just like the researchers who studied the alien device, cognitive scientists ask what motion features create a strong impression of animacy. Some researchers provided demonstrations with hand-tailored stimuli and then went on to adjust the features of these to see what features are necessary (Bloom & Veres, 1999; Klein et al., 2009). Other researchers investigated possible motion primitives (Tremoulet & Feldman, 2000; Tremoulet & Feldman, 2006). The research has been criticized on the response side Gao, Newman & Scholl (2009); Scholl & Gao (2013). In particular, Gao, Newman & Scholl (2009) pointed out that asking subjects to rate the animacy of displays is problematic as the subjects would attempt to give some plausible animacy rating, even though they did not found any of the particles displayed in Tremoulet & Feldman (2000) particularly animate. Their judgments may thus reflect the task demands rather than attributes of human perception. With regard to the stimuli, Roux, Passerieux & Ramus (2013) showed that a difference in the total fixation time dedicated to a pair of moving objects in two different hand-tailored displays used in Klein et al. (2009) disappears once the distance between the two objects is controlled. Finally, researchers offered computational level analyses (Feldman & Tremoulet, 2008; Baker, Saxe & Tenenbaum, 2009). These analyses first formalize the task of an observer. They then derive the optimal solution for this task and test whether human behavior corresponds to the optimal solution.

The current study investigates what motion features are responsible for the perception of goal-directed motion and chasing in particular. The study focuses on the reverse-engineering of the search process. In this respect, the current study has a similar goal as the studies that formally model the perception of goal-directed motion (Feldman & Tremoulet, 2008; Baker, Saxe & Tenenbaum, 2009; Tauber & Steyvers, 2011). However, we pursue a different approach. The computational-level models and their Bayesian implementation (Baker, Saxe & Tenenbaum, 2009) in particular have been criticized for the lack of performance-level predictions (McClelland et al., 2010; Jones & Love, 2011). These models predict what tasks and stimuli people master but they don’t tell us how people do it. In the context of the perception of goal-directed motion, the model in Baker, Saxe & Tenenbaum (2009) tells us what goals and future actions people predict, based on an observation of the agent’s motion, but it doesn’t tell us how people make these inferences. How are the cues to goal-directed motion extracted and processed? Which aspects of the agent’s motion are focused by the observer? What parts of the display are surveyed? This kind of focus isn’t problematic in itself. Rather, it constrains the number of predictions these models can provide and hence constrains their explanatory power (Jones & Love, 2011; Bowers & Davis, 2012).

The current study pursues a different approach to reverse-engineering, which is popular in psychophysics. Rather than focusing just on the overall performance (e.g., chase detection accuracy) and the factors that affect it, we investigate several intermediate events of the search process and how they affect the final detection. The current study presents a first stage in this effort. We decompose the search process into distinct events such as saccades or pursuit eye movements. We describe these events in terms of stimulus properties that trigger and accompany the events. With such description at hand, one should be able to set up a computational model that can simulate the human search process. In the next two sections, we sketch two methodological elements that will be crucial for the first exploratory stage.

### Template analysis

To return to the story of the alien device, consider the critics who argued that one-by-one trial-and-error identification of features is inefficient and susceptible to bias since there is no rule that will tell the researchers which features to select for the next investigation, such that the feature space is fully explored. This group proposed an alternative approach. They proposed to present displays of random noise to the device. Then, they argued, one just needs to look at the noise patterns that are followed by a strong device response and these will tell us what kinds of motion the device prefers. No prior hypothesis about the candidate motion pattern or the underlying primitives is required.

A similar approach has been pursued by neurophysiologists. Neurophysiologists asked what kind of stimulus triggers the strongest neural response. You may recognize that this problem is rather similar to the one faced by the researchers studying the alien device. Following a hypothesis-driven strategy, one would tackle the question by measuring a neuron’s response to vertical lines, T-shaped edges, letters of the alphabet, ellipse shapes, heart contours, photographs of human faces and so on. There is an infinite number of possibilities. Neurophysiologists developed a more efficient strategy to pin down a neuron’s preferred stimulus pattern (Ringach & Shapley, 2004). They measured the neuron’s response to an array of images which show random noise. They then analyzed the relationship between the neuron’s response and the properties of the stimulus. The simplest case of such an analysis is to average all images that are followed by a neural response to obtain a template image. Another template image is obtained by averaging all images that were *not* followed by a neural response. Finally, by taking the difference between the two templates one obtains an image that describes the neuron’s response and, as such, has been dubbed *receptive field*. This image provides a rule to classify the stimuli and hence to predict the neuron’s response. For instance, Ringach, Hawken & Shapley (2002), showed that the receptive fields of neurons in the macaque primary visual cortex are well described by two-dimensional Gabor functions. Neurons with such receptive fields are good at edge detection.

This procedure from neurophysiology has been adapted in psychophysics. Instead of measuring the neuron’s response, the psychophysicist derives a template image based on the subject’s behavior. For instance, Solomon (2002) asked subjects to detect a Gaussian blob in noisy images. The obtained difference image indicated Gabor-based edge detectors. These difference images have been labeled *classification images* (Eckstein & Ahumada, 2002; Victor, 2005) or (in analogy to receptive fields) *perceptive fields* (Neri & Levi, 2006; Kienzle et al., 2009). In this report, we refer to them as classification images. There are multiple methods to compute classification images. Reverse-correlation has been the most popular procedure (Neri & Levi, 2006), but alternative classification techniques from statistics and machine learning have been proposed and applied (Victor, 2005; Kienzle et al., 2009). In this report, we will collectively refer to any method that produces template or classification images as *template analysis*. As pointed out by Neri & Levi (2006), transferring this approach from physiology to psychophysics involves some difficulties: “[...] One can simply measure the response of a neuron to pure noise, but if a human subject views a display consisting only of noise, it is unlikely that he or she will generate coherent responses. At the very least, the experimenter is required to specify a task to be performed, and tasks typically relate to the presence or absence of a signal that is added to the noise on some trials.” (p. 2466) Once the experimenter needs to select a signal/target and the task, the results of template analyses become contingent on this decision and may reflect the experimenter’s stimulus choice rather than any aspect of cognition.

One way to resolve this uncertainty is to compare the subject’s template and classification images with those of an ideal observer (e.g., Solomon, 2002). An ideal observer is a hypothetical subject who maximizes her performance with respect to the feedback. If the subject’s performance diverges from that of an ideal observer, then factors other than the experimenter’s choices of the stimulus and the task constrain the behavior. If the performance of the subject and the ideal observer coincide, then it’s possible that the experimenter’s task and stimulus choice, rather than the cognitive factors constrain the behavior.

Several recent studies used detection tasks to study human perception of goal-directed motion (Gao, Newman & Scholl, 2009; Gao & Scholl, 2011; Meyerhoff, Schwan & Huff, 2014a; Meyerhoff, Schwan & Huff, 2014b). These studies focus on chasing as a case-study. Chasing motion is created by letting a chaser move towards a chasee who moves randomly. Randomly moving distractors are added to the display and the subject is asked to detect the chasing pair. One can perform template analysis by comparing the trials where the subject responded “chase present” to those where she responded “chase absent.” One needs to consider that unlike all of the previously cited template analyses, the studies of chasing use time-varying stimuli. As a consequence, the corresponding template and classification results will be movies rather than images. As another consequence, the search task where the subjects terminate a trial used by Meyerhoff, Schwan & Huff (2014b) is better suited than the two alternative forced-choice task used by Gao, Newman & Scholl (2009), in which the trials have a fixed length. The former allows the subject to accurately pin down the time when the critical motion features occurred.

### Event decomposition based on the eye movements

Another consequence of dynamic stimuli is, that in order to inspect the agents’ properties, the subjects need to track the agents with their gaze. Similar to studies that use template analyses (e.g., Ringach & Shapley, 2004), one could simply ask the subject to fixate a cross at the screen center with their gaze throughout a trial. With a fixated gaze, one needs to assure that the candidate motion is presented not far away from the fovea. However, in a typical chase detection task (Gao, Newman & Scholl, 2009; Gao & Scholl, 2011; Meyerhoff, Schwan & Huff, 2014a; Meyerhoff, Schwan & Huff, 2014b) the candidate identification is a logical part of the search process. The gaze control thus removes parts of the phenomenon we wish to study. Instead, the subjects are allowed to perform eye movements, and the gaze location at the time when the subject responds is measured with an eye tracker. The gaze location is then used as a positional marker to align samples in the template analyses. In addition, if the gaze is in motion, one can use the gaze direction to align the samples with the direction of the gaze movement. Such a combination of eye tracking and template analyses is not unprecedented. Kienzle et al. (2009) used saccade targets from a free viewing task to positionally align the samples in their template analyses.

Let us briefly review the eye movement literature to see what gaze events can be used to decompose the search process and to define the samples for template analyses. When tracking dynamic stimuli, people perform pursuit eye movements (Barnes, 2008). Pursuit eye movements help to stabilize targets at the center of the fovea, allowing a closer inspection (Schütz et al., 2008) and prediction of future motion (Spering et al., 2011). Pursuit eye movement consists of smooth eye movements and catch-up saccades. Smooth eye movements help to align the gaze with a smoothly moving target. During smooth eye movements, the gaze velocity matches the target velocity with a gain (which is the gaze velocity divided by the target velocity) between 0.9 and 1 (Meyer, Lasker & Robinson, 1985). When the target velocity and acceleration can’t be matched by a smooth eye movement (such as during target’s instantaneous direction change), catch-up saccades are necessary to realign the target at the center of the fovea. Since the target is already located in the vicinity of the gaze, catch-up saccades are usually short (De Brouwer et al., 2002). Catch-up saccades are followed by smooth eye movements where the initial position, velocity, and direction of the gaze closely matches the position, velocity, and direction of the target (Lisberger, 1998). Catch-up saccades should be contrasted with more typical saccades that follow attention relocation (Kowler et al., 1995). We will refer to the latter as exploration saccades. The two types of saccades can be distinguished based on the precision (catch-up saccades match better the position of the target and enhance the gain) and based on the fact that catch-up saccades are preceded and followed by smooth eye movements.

We focus on four types of gaze events: exploration saccades, catch-up saccades, smooth eye movements and the final detection. We use gaze location and the event’s start/end to determine the samples for the template analyses. The template analyses are used to describe what motion features attract the saccades, what motion features are focused during smooth eye movements, and what motion features qualify the pursued candidate for the detection.

To obtain robust results with the template analyses, it is essential to gather a large number of samples with a good signal-to-noise ratio. As the purpose of the current study is exploratory, we decided to sacrifice the generality of the conclusions and instead we collected a large number of trials from few subjects. As a consequence, the result presentation is descriptive and the inferences are being made on a per subject basis. In our opinion the current approach nevertheless provides a valuable insight into the process that facilitates the detection of chasing. The current approach complements the literature on perception of goal-directed motion that has been dominated by a hypothesis-driven research so far.

## Methods

### Subjects

Four subjects (one male) were recruited among the students of the University of Heidelberg. The subjects’ age ranged between 23 and 28 years. All subjects had normal or corrected-to-normal vision. The study consisted of six sessions. Each session took from 60 to 90 min. Each subject received 60 Euro. At the start of the first session, each subject provided a written consent regarding their participation. The subjects were also asked whether they agree with the publication of the obtained data. All subjects agreed. The ethics committee of the Faculty of Behavioral and Cultural Studies of the University of Heidelberg provided a statement that there are no ethical concerns or objections with respect to the choice of goals and design, choice of human participants, or the measures used in the current study. The statement was provided as a letter from the ethics committee to the second author on 27 March 2012.

### Stimulus

The subjects watched 14 white rings (1 degree diameter) moving on a gray background on a monitor. One of the rings was selected as the chaser and another as the chasee. The remaining rings served as distractors. All rings moved with a constant speed of 14.5 degrees per second. All rings made 5.4 direction changes per second, on average. For the chasee and the distractors, the new motion direction was selected from a uniform continuous distribution ranging from 60 degrees to the left to 60 degrees to the right of the old movement direction. The chaser always changed its motion to head directly towards the chasee in a heat-seeking manner. The motion of the rings was confined to an invisible square area of size 26 × 26 degrees. This meant that the rings would sooner or later hit an invisible wall. The chasee and the distractors bounced off the wall (i.e., the movement direction was symmetrically mirrored around the normal of the wall). Upon touching the wall, the chaser moved towards the chasee. The rings were pervasive to each other—there were no collisions among the rings. Rejection sampling was used to constrain the minimum chaser-chasee distance to 3 degrees. The area size and the number of agents was chosen such that the average distance from a distractor to its nearest neighbor is the same as the average distance between the chaser and the chasee. Since the minimum chasee/chaser distance was mostly also their average distance, the subjects may use the 3 degrees distance as a detection rule. To avoid this, 4 out of 40 trials did not use rejection sampling and the minimum distance was not constrained. We label the former distance-constrained (DC) trials. We label the latter non-constrained trials (NC).

### Procedure

The experiment was divided into six sessions. Each session consisted of 3 to 4 blocks with short breaks between the blocks. A block consisted of 40 trials. All subjects saw the same trials. However, the trial order in each block was randomized across the subjects. At the start of the first session the subjects read an instruction which explained the task. In particular, the subjects were asked to press the mouse button as soon as they detected two chasing agents. The experiment was performed in a dimly lit room. Subjects were seated 50 to 60 cm from a CRT screen run at 85 Hz. All values in degrees of visual angle are computed for a 50 centimeters distance. At the start of the first session, the subjects completed ten training trials.

We measured the eye movements with an Eyelink II Eyetracker run at 500 Hz in a fovea-based mode. The Eyetracker was calibrated before each block. Subjects were asked to initiate each trial by looking at a fixation cross which appeared at the center of the screen. The presentation software checked the distance from the subject’s fixation point to the screen center. If the distance was smaller than 3 degrees, a correction was performed that shifted the gaze to the screen center. If the distance was larger than 3 degrees, the measurement was repeated few times. If after 0.5 s from the initial measurement the measured gap was still more than 3 degrees a new calibration was performed.

A trial was terminated either with a button press, or it ended automatically after 30 s of no response. After a button press, the rings instantly stopped moving and remained frozen at their last position. The mouse cursor appeared and the subject was asked to select the two agents that she had detected. If both, chaser and chasee were detected, we counted the trial as success. After 10, 20, 30 and 40 trials the subjects were shown the proportion of correct trials on the last ten trials. PsychoPy (Peirce, 2007) was used to control the experiment. Materials, data and programming code used in this project are available from http://github.com/simkovic/Chase/releases/tag/peerjsub.

### Pre-processing of eye-tracking data

The data from each eye on each coordinate axis were separately smoothed by a Gaussian filter with a standard deviation of 20 ms. Missing data from episodes shorter than 100 ms were linearly interpolated. In most cases, the data from both eyes were available and we averaged them to obtain the gaze position. If the distance between the coordinates of the two eyes was larger than 4 degrees, the gaze was flagged as missing. After smoothing, the gaze position was linearly interpolated to 85 Hz (the monitor frame rate). Next, we extracted four *basic events* based on the gaze velocity, gaze acceleration and the minimum event duration. The four events were *fixations*, *saccades*, *slow smooth movement* (SSM) and *fast smooth movement* (FSM). Fixations and saccades were extracted based on the thresholds listed in Table 1. The identification of smooth eye movements was based on the following consideration. The velocity of smooth eye movement matched the object velocity or was slightly slower. The velocity of a smooth eye movement was not constant but showed oscillations with an amplitude up to 5 degrees per second (for an example, see the right lower panel of figure 1 in Barnes, 2008). We chose a velocity boundary for FSM that is centered around 14.5 but provides large enough margins so that oscillations are not flagged as saccades. For FSM, in addition to the criteria in Table 1, at least one agent with the following properties was required. During the first 100 ms the average gaze-agent distance had to be less than 1 degree. During the whole event, the average gaze-agent distance had to be less than 4 degrees. Finally, the median angular absolute difference between the motion direction of the gaze and the agent had to be less than 25 degrees. When a smooth movement is not preceded/followed by catch-up saccades, its initiation and termination is not instantaneous but it rather ramps up/slows down gradually. Furthermore, a brief look at the data showed that the subjects often tracked groups of multiple agents with a strategy similar to the one described in Fehd & Seiffert (2010). The subjects pursue groups of agents with the gaze at the group center. When tracking the group center, the smooth movement would not be identified as FSM, because the gaze would not be aligned with any particular agent. To correctly classify such cases we defined slow smooth movement (SSM). To qualify as SSM, the criteria in Table 1 had to be satisfied and the event should not qualify as FSM. In addition, the criteria determined which agents were in focus during fixation, SSM and FSM. For FSM, these were agents that satisfied the above stated criteria. For SSM and fixations, these were all agents whose average distance to the gaze was less than 4 degrees.

**Table 1:** Thresholds for identification of basic events. The numbers in each cell show the maximum and/or minimum.

Basic event	Velocity deg/s	Acceleration deg/s^2^	Duration ms
Fixation	[0, 6]	<800	>80
Slow smooth motion	[4, 21]	<800	>80
Fast smooth motion	[9, 21]	<800	>100
Saccade	>21	>4000	>20

Next, we extracted complex events: *pursuit* and *exploration*. The complex events separated the gaze data into periods when different agent sets were focused. Pursuit consisted of smooth movement and fixations. Exploration consisted of fixations and SSMs that did not qualify as pursuit. Exploration was used to rapidly move the gaze across the screen in order to identify candidates for pursuit. Pursuit allowed closer inspection of groups of agents.

The identification of exploration and pursuit was automated as follows. Pursuit started either with FSM or with two consecutive SSM events (separated by a saccade) that focused the same agent(s). A pursuit event continued as long as the agent sets between the consecutive basic events (fixations, SSMs and FSMs) overlapped. Finally, each pursuit event had to include at least one FSM. The idea behind this algorithm was to use FSMs to reliably and conservatively identify the catch-up saccades. Catch-up saccades then determined exploration saccades as their complement. The start and end of the saccade determined the start and the end of the samples for the template analysis.

Unfortunately, the human eye movements proved to be too variable and the algorithm produced systematic misclassification. In particular, the subjects would pursue rings that were further apart than 4 degrees. If the subject’s catch-up saccades alternated between the two agents, the algorithm would not detect a single pursuit event but would instead separate it into a mix of exploration and pursuit events. We therefore surveyed and corrected the results of the automated complex event extraction manually. In addition, the coding process allowed us to identify and repair, or to exclude trials with bad calibration or with large chunks of missing data. The event survey was done with the help of a custom software written in PsychoPy that displayed the rings and the gaze point as well as velocity, acceleration and basic and complex events. The software tool allowed the coder to interactively create, delete, and alter the complex events produced by the algorithm. The GUI can be seen in Movies S1, S2, S10, S12 and S13. The column on the right shows velocity, acceleration, fixations, saccades, SSM and FSM events. The row at the bottom shows saccades in blue and the pursuit events in red. Displayed are the final, manually corrected pursuit events. Agents are drawn as white circles. The gaze point is shown as a black circle. The pursued agents are highlighted in color. When we refer to a particular time point in a movie, we mean the trial time displayed by the counter below the circles. For instance, Movie S1 shows pursuit where the agents are further than 4 degrees apart at time 3–4 s after the trial onset. In Movie S1 there is only a single pursuit event. Movie S2 shows a more typical example, with multiple pursuit events.

Using the GUI, the first author surveyed all subjects. A second coder who was naive to the research question was given a written protocol that described her task. The protocol is included in the repository. The second coder surveyed subject 1 and 2 and her coded events were integrated with that of the first coder. Table 2 lists various inter-rater reliability measures computed by comparing the results from the first rater against the results from the second rater. The last column shows good reliability with all correlation coefficients *r* > 0.63. We further discuss the validity of the event identification procedure in the penultimate section of general discussion.

**Table 2:** Inter-rater reliability of event identification. Inter-rater reliability of the first coder computed against the second coder. Correlation in the last column was computed with Matthews correlation coefficient.

Event	Accuracy	Sensitivity	Specificity	Correlation
CS vs. ES	0.89	0.86	0.92	0.78
ES vs. CS	0.89	0.92	0.86	0.78
CS1 vs. rest	0.90	0.69	0.94	0.64
CS1 vs. ES	0.89	0.69	0.97	0.72

### Analyses

**Figure 1:**
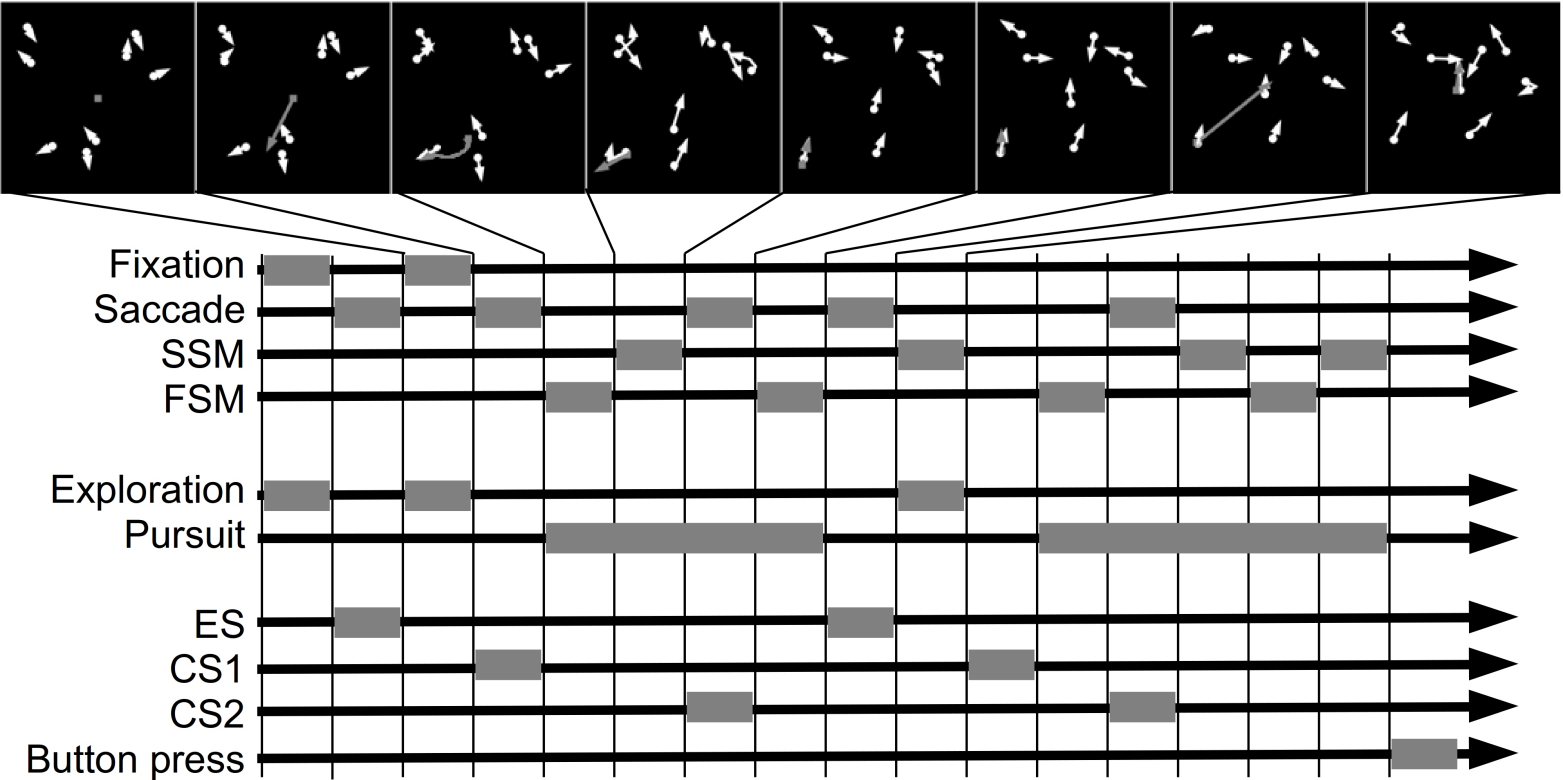
Example of basic and complex events. The panels in the top row show snapshots of the search behavior. The gaze position is shown by the gray circle. Agents are shown as white circles. The movement trajectory is indicated by the corresponding arrow. The diagram below shows basic and complex events as well as exploration and catch-up saccades. Each gray block shows when an event occurred. The time flows from the left to the right. To improve the readability of the figure, all basic events are equally long.

We identified saccades that initiated and maintained the complex events. In particular, we labeled as *exploration saccade* (ES) a saccade that was immediately followed by exploration. We called a saccade, that was immediately followed by a basic event which belonged to pursuit, *catch-up saccade* (CS). Furthermore, within each pursuit event we distinguished the order of the saccades. We distinguished the *first catch-up saccade* (CS1), the *second catch-up saccade* (CS2) and so on. The target location and the time at saccade onset defined the samples that were used to compute the template and classification results. Figure 1 illustrates, on a doctored example, how basic and complex events determine the position of exploration and catch-up saccades.

The total number of samples used in the template analyses are listed in Table 3 for each subject. Since the samples show directed motion it was necessary to rotate the samples such that the direction of their motion is aligned. In cases where the gaze was moving (such as during pursuit) the samples were aligned with respect to the gaze direction. In cases where the direction of gaze was undefined (such as after ES which were often followed by a fixation) the samples were aligned with respect to the agents’ dominant movement direction. We determined the dominant direction as the mode of a radial histogram of the agent’s motion direction on consecutive frames (sampled at 85 Hz). The histogram was computed over all frames in the sample time window and over all agents. To ensure that only agents in vicinity of the positional lock contributed to the mode computation, the agents were weighted based on their distance to the positional lock with a circular polynomial window of radius 5 degrees.

**Table 3:** Number of samples for each event and each subject.

Subject	D	ES	CS1	SME
1	737	10,760	3,580	14,655
2	769	8,272	3,566	12,741
3	842	11,859	4,271	17,101
4	831	7,606	2,467	7,495

**Notes.**

D detection ES exploration saccade CS1 first catch-up saccade SME smooth movement episode

To obtain template movies, averaging and principal component analysis (PCA) were used. The advantage of averaging is that it can be applied to samples with variable length (such that at a later time point in the template fewer samples contribute to the average). The disadvantage of averaging is that it may produce artifacts when the pixel distribution is not unimodal. For instance, if one obtains an average template with two agents—one before and one behind the gaze, the researcher may conclude that most samples include two agents. However, it may be equally the case that in half of the samples the subject pursued a single agent aligned behind the gaze point and in the other half she tracked a single agent but the gaze point was locked in front of the agent. If such samples were averaged together, one would obtain a template with two agents even though none of the samples featured two agents. PCA is able to identify such cases as separate principal components. If the distribution is unimodal, the average template will correspond to the first principal component.

PCA was computed by eigenvalue decomposition (see chapter 12.1.4 in Bishop, 2006, for details). Average templates were computed with non-parametric regression (Wasserman, 2004, section 20.4). Non-parametric regression works with the coordinate representation of the samples. This is computationally more efficient than first rendering the samples as short movies and then averaging over these. The motivation and the computation behind the non-parametric regression are further explained in section 2 of Supplemental Information 1.

Similar to Kienzle et al. (2009) we adapted support vector machines to compute classification movies. The analyses mostly followed those of Kienzle et al. (2009). The details are provided in section 1 of Supplemental Information 1.

The results of the template analyses are reported as supplemental movies in AVI format (e.g., Movie S3). Note that the animations are graphs. The resolution of each template (in pixels) and the number of frames reflect the decisions made in the analysis. To facilitate the relative orientation, each movie contains a time bar. The movie time does not correspond to real time. To find out what the ticks on the time bar correspond to in absolute terms—in seconds, please look at the definition of the time window and the time lock in the text. The time lock is either at the start, in the middle or at the end of the time window. With few exceptions the time window is 0.8 s long which translates into 68 frames (at 85 Hz). To facilitate comparison, each movie shows multiple templates positioned on a grid. Usually, each row shows a single subject. The precise details about what each panel shows are provided in the figure description. In many cases, the patterns in the template movies concerned only the axis with respect to which the samples had been aligned (i.e., gaze direction or dominant motion direction). To simplify the presentation of results, we discarded the axis orthogonal to the alignment axis. We plotted the pixel values (that are averaged over a sleek band of 0.3 degrees around the alignment axis) as a function of the position at the alignment axis and the position in time. These plots make it possible to follow most of the result exposition without having to switch to the movie material. Constant agent motion manifests in these diagrams as a line with slope equal to the agent’s average velocity. The chase pattern manifests as a pair of parallel lines (e.g., S2 in Fig. 4), which look similar to rails of a railroad track. For the remainder of this report, we will refer to these diagrams as *rail diagrams*. The cell layout of a rail diagram usually follows the cell layout of the corresponding movie.

We further analyzed the maxima in each rail diagram by fitting one or two lines to the graphs. The shortest distance of a point (*x*, *t*) (with *x* the position on the alignment axis and *t* the time) in the rail diagram to a line is given by *h*(*x*, *t*) = (*p* + *vt* − *x*)/(1 + *v*^2^), where *p* is the offset and *v* is the slope of the line. The fitting was done with simplex algorithm (Nelder & Mead, 1965) by maximizing the correlation between the rail diagram normalized to the range [0, 1] and a pattern that was obtained by summing two (or one in some cases) uni-modal functions. The value of the uni-modal function at *x*, *t* was $\max (0,1-{\left[h(x,t)/s\right]}^{3})$ where *s* is another free parameter that determines the width of the maximum. This function was hand-tailored to match the functional shape of the mode in the rail diagram and to return values in the range [0, 1]. The estimates of *p*, *v* and *s* are listed in Table 4.

**Table 4:** Estimates of parameters that describe the lines fitted to the rail diagrams.

Event	Subject	*p*_1_	*v*_1_	*s*_1_	*p*_2_	*v*_2_	*s*_2_	*p*_1_ − *p*_2_
D	S1a	0.52	−11.48	0.28	−3.20	−11.02	0.09	3.72
	S1b	3.08	−12.92	0.11	−0.01	−12.82	0.22	3.08
	S1	0.09	−11.75	0.37	–	–	–	–
	S2	2.64	−13.37	0.16	−1.02	−13.47	0.16	3.66
	S3	2.19	−13.21	0.16	−1.33	−13.02	0.17	3.52
	S4	2.11	−13.74	0.16	−1.69	−13.06	0.15	3.79
ES	S1	−0.14	−13.31	0.36	–	–	–	–
	S2	0.10	−13.38	0.35	–	–	–	–
	S3	−0.07	−13.32	0.35	–	–	–	–
	S4	0.15	−12.49	0.44	–	–	–	–
CS1	S1	−0.64	−13.63	0.33	–	–	–	–
	S2	−0.48	−13.32	0.35	–	–	–	–
	S3	−0.64	−13.40	0.34	–	–	–	–
	S4	−0.23	−12.97	0.42	–	–	–	–
IO	–	–	−13.99	0.11	–	−13.93	0.10	4.21

**Notes.**

*p* offset at *t* = − 0.2 s in degrees *v* slope in degrees per second *s* band width of the maximum in the direction orthogonal to the line D detection event IO ideal observer

## Detection time and detection accuracy

### Results

Eye-tracking provides a more fine-grained measure than detection time and accuracy. Nevertheless, for the comparison with exclusively behavioral experiments, it can be helpful to investigate detection times and accuracy.

**Figure 2:**
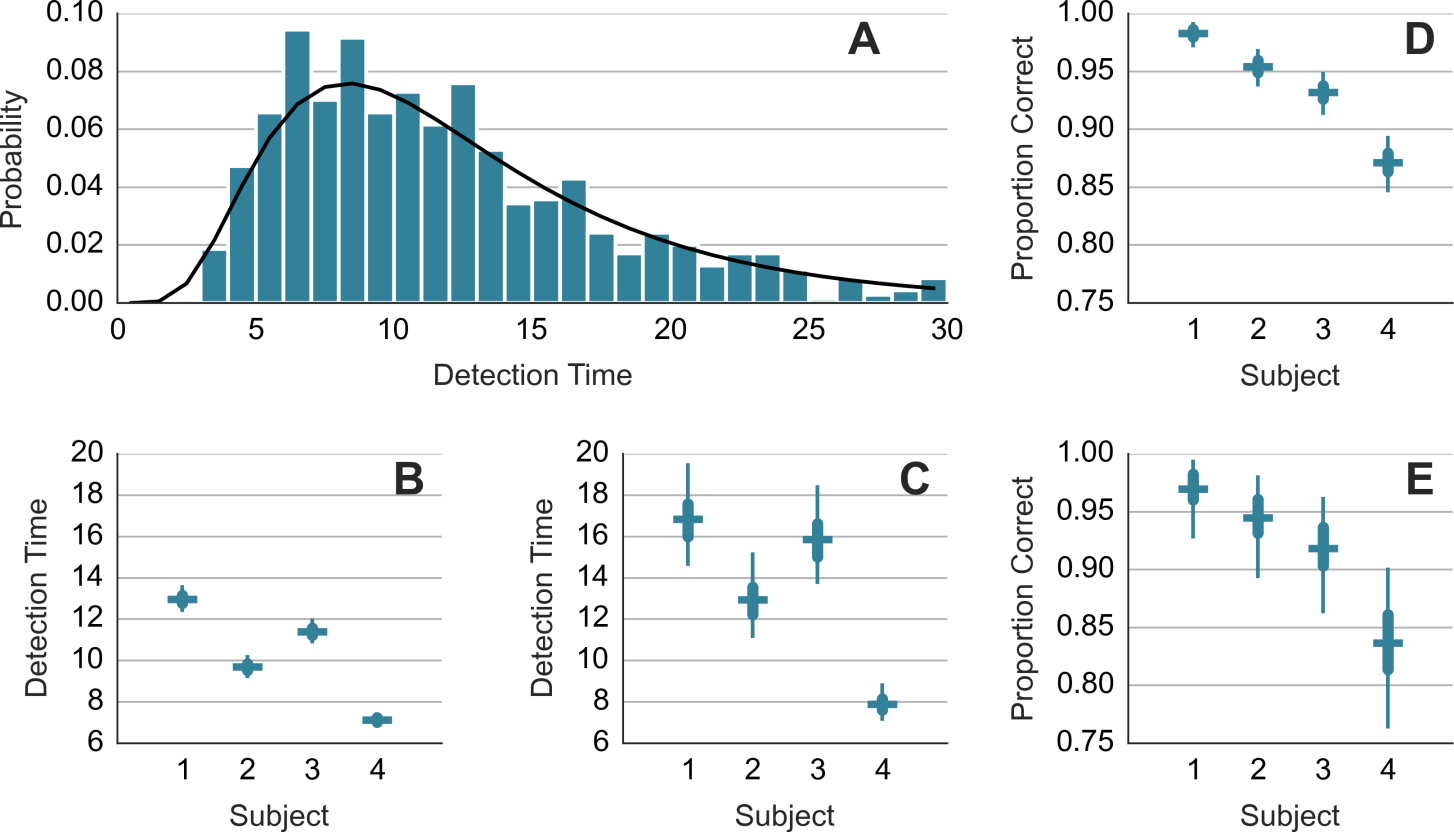
Detection time and detection rate. (A) shows histogram of the detection times of subject 1 along with the probability density function of the fitted model. (B) and (C) show mean response time. (D) and (E) show detection rate. (B) and (D) show results from trials where the minimum distance was constrained. (C) and (E) show results from trials where the distance was not constrained. Each error bar shows the median (horizontal line) of the estimate along with the 50% interval (thick vertical line) and the 95% interval (thin vertical line). Note that mean detection time is a random variable and the median and other percentiles are used to describe this random variable.

For each subject, a log-normal model was fitted to the detection times *y_t_* on trials *t* = 1, 2, …: $\log ({y}_{t})\sim \mathcal{N}(\mu ,\sigma )$ where *μ* and *σ* were the estimated parameters. The fitting was done with STAN (http://mc-stan.org). A separate model was fitted to the detection times from the trials in which the chaser-chasee distance had been constrained (DC) and trials where it had been not constrained (NC). Figure 2A shows the normalized histogram of the detection times and the obtained fit (black curve) for DC trials of subject 1. Figures 2B and 2C show the mean detection times exp(*μ* + *σ*^2^/2) for DC and NC trials, respectively. The detection takes 2–4 s longer in the less frequent NC trials. We modeled the detection successes with Bernoulli distribution. We estimated the value of the probability parameter for each subject separately. We refer to the probability parameter as the detection rate. Figures 2D and 2E show the detection rate for DC and NC trials. Subjects were less accurate in the NC trials. Due to a smaller sample size, the NC estimates are wider. Comparison across subjects suggests a trade-off between speed and accuracy. Subject 1 is accurate but slow. Subject 4 is fast but less accurate.

**Figure 3:**
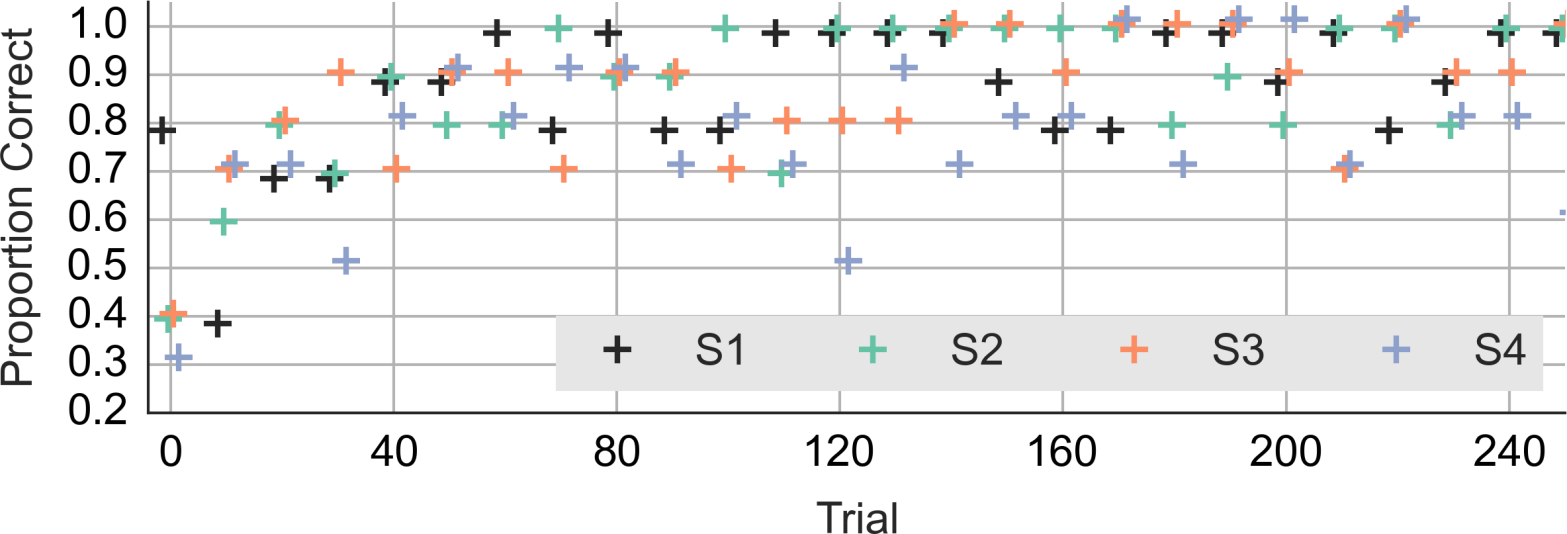
Accuracy during the first 240 trials. The vertical axis shows the accuracy feedback provided every ten trials. The horizontal axis shows consecutive trials. S1, S2, S3 and S4—data from the four subjects.

Did the accuracy change across trials and blocks? Figure 3 shows the performance during the first 240 trials. It shows the averages for consecutive ten-trial sets. This was the feedback that subjects saw during the experiment. At the start of the experiment, the subjects got only 3 or 4 out of ten correct. Even subject 1, who had scored 8 out of 10 correct on the training trials, lapsed to 40% correct in the first block. However, all subjects did quickly improve to around 90% correct.

**Figure 4:**
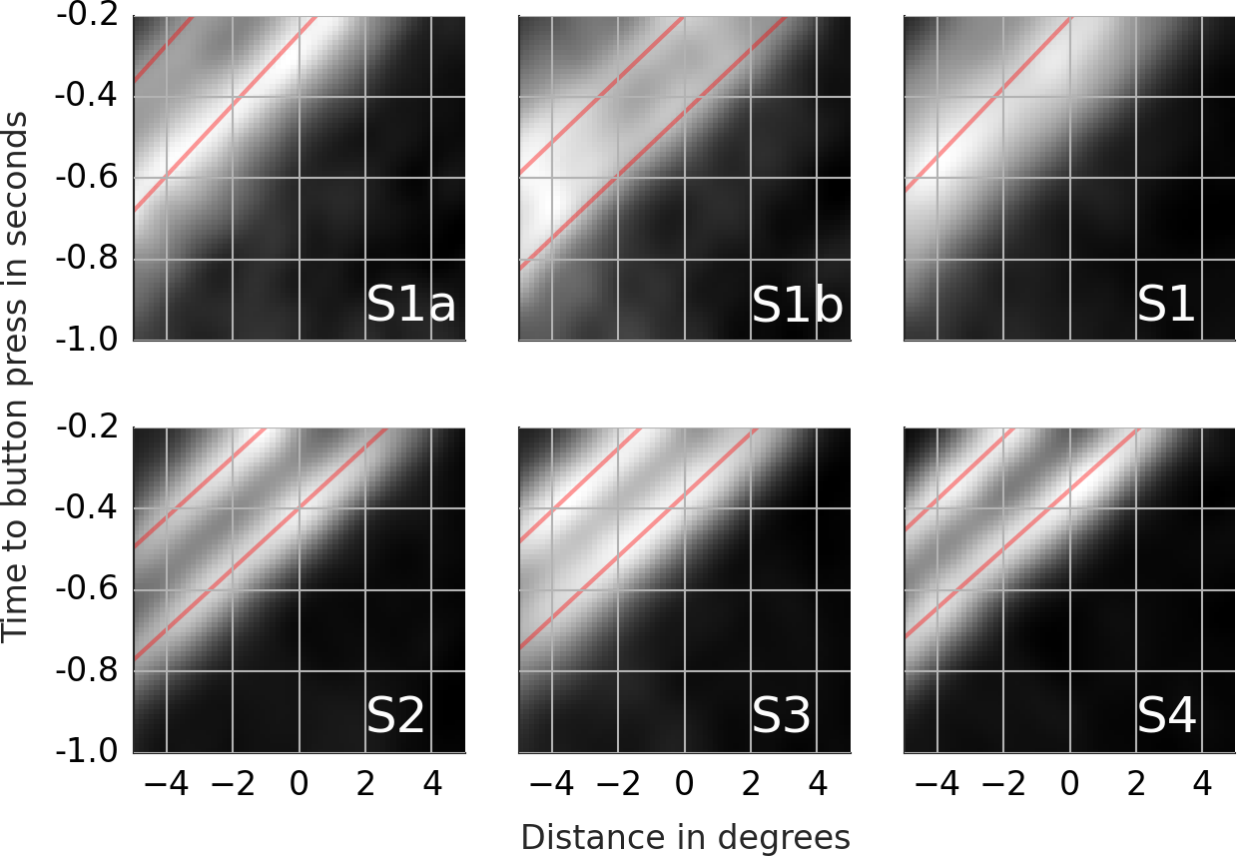
Rail diagrams of the first principal component of the detection events. S1a and S1b show respectively PC1 + PC2 and PC1 − PC2 of subject 1. S1, S2, S3 and S4 show PC1 of subject 1, 2, 3 and 4. The vertical axis shows time with respect to button press in seconds. The horizontal axis shows the distance from the positional lock in degrees. Agent’s motion manifests as an elongated maximum where the slope along the elongation can be interpreted as the agent’s velocity. To estimate the mean velocity, lines (red) were fitted to the maxima.

### Discussion

We compare the current results to the results in Meyerhoff, Schwan & Huff (2014b) who used a similar self-paced chase detection task. The mean response time in Meyerhoff, Schwan & Huff (2014b) (see the twelve-objects condition in Fig. 6) is approximately 7.5 s. Subjects 1, 2 and 3 in the current study are considerably slower. The mean accuracy in Meyerhoff, Schwan & Huff (2014b) is approximately 0.7. The subjects in the current study are more accurate. We think that this difference is due to a different maximum trial length and different instruction. Meyerhoff, Schwan & Huff (2014b) put more emphasis on a quick response. In Meyerhoff, Schwan & Huff (2014b), the maximum trial length was 15 s and if subjects provided no response a message saying, too slow” was displayed. In the current study the maximum trial length was 30 s and there was no feedback when an omission happened (However, omissions were counted as incorrect responses and even though the feedback was stochastic it is possible that the subjects noticed this fact at some point during the experiment and tried to avoid omissions.).

Did the inclusion of the marking procedure at the end of each trial during which subjects selected chaser and chasee affect the response time? It is possible that the marking procedure required additional memory resources which resulted in prolonged response times. However, we do not think this is the case. The only cues to the agent’s identity were its position and direction of motion. Both these cues were accounted for by the eye movements. At the end of each trial, the subject did not need to fully recall which agents she was tracking. Simply marking the two agents nearest to where her gaze ended after the motion stopped would be sufficient. An exception were trials in which the tracked agents crossed their trajectories and sometimes overlapped with other agents. In these instances, the subjects may prefer to prolong tracking until the tracked agents reach a position where there is no overlap. However, with agents moving at 14.5 degrees per second, it would take only half of a second at most for the tracked agents to move into empty space.

## Chase detection

### Results

Most of the trials ended with a button press that signaled that the subject had found the chasing pair. Shortly before the button press, the subject must have spotted some, possibly crucial, piece of evidence that triggered the final decision. It takes at least 100 ms from the decision to the motor response (Kornhuber & Deecke, 1965). There is likely an additional lag due to decision-making. We set the time window from 1,000 ms to 200 ms prior to the button press with time lock at 200 ms. The samples were locked at the position where the gaze was at the time lock. The samples were analyzed with PCA. Movie S3 shows the first ten principal components (columns) for each subject (rows). The first row of Table 5 lists the proportion of variance explained by the first five components. The rail diagrams of PC1 are shown in Fig. 4 S1–S4. The lines that were fitted to the maxima in the rail diagrams are shown in red. Table 4 D lists the best-fitting line parameters.

First, consider subjects 2, 3 and 4. The first principal component (PC1) of these subjects shows two agents moving horizontally one after another in the same direction. PC1 explains 6.8 to 8.0 percent of variance. The second and third component shift the first component vertically and horizontally. This can be done by adding PC2 or PC3 to PC1. If one instead subtracts the components, then PC1 is shifted in the opposite direction. This is shown in row A of Movie S4. PC2 of subject 2 in the second panel was subtracted from PC1 in the first panel (from left). The third panel shows the result. A finer shift can be obtained by adding a ternary (or higher order) shift pattern such as PC5 of subject 4. When comparing the PCs across the subjects, it is important to keep in mind that formally each PC is an eigenvector and thus defined up to scale. Even though some PCs are inverted between the subjects (compare PC2 of subject 2 with PC2 of subject 3), they show identical eigenvectors. For subjects 2, 3 and 4, the agents’ velocity is 13 to 14 degrees per second (Table 4D). 200 ms before the button press, the chasee is located 2.1 to 2.7 degrees in front of the gaze while the chaser is located 1 to 1.7 degrees behind the gaze. The distance between the agents is 3.5 to 3.8 degrees.

The rail diagram of the PC1 of subject 1 (S1 in Fig. 4) shows a single maximum that is stretched out horizontally and that moves with speed of 11.75 degrees per second. However, PC2 to PC5 indicate two agents and are similar to PCs of subjects 2, 3 and 4. By adding PC2 to PC1 from subject 1 one obtains a pattern that is similar to PC1 of subjects 2, 3 and 4. This is shown in row B of Movie S4. On the other hand, by *subtracting* PC2 from PC1 one obtains another chase pattern where the gaze is focused on the chaser instead of the chasee (not shown). The rail diagrams of PC1 − PC2 and PC1 + PC2 of subject 1 are shown in Fig. 4 S1a and S1b and the results of line fitting are included in Table 4. Since the templates resulting from the combination of PC1 and PC2 of subject 1 show a slight vertical offset, we averaged the second and third pixel row above and the second and third pixel row below the central axis for PC1–PC2 and PC1 + PC2, respectively. Similar to subject 2, 3 and 4, subject 1 focuses two agents. Unlike the remaining subjects, subject 1 aligns her gaze with individual agents. In S1b the gaze is located at the chaser’s position. In S1a the subject focuses the chasee with gaze 0.5 degrees behind the agent.

### Discussion

Why did PCA return the PC1 and PC2 decomposition rather than PC1 − PC2 and PC1 + PC2? The components of PCA are by definition orthogonal. However, the two patterns PC1 − PC2 and PC1 + PC2 are correlated, and hence it was not possible to obtain this decomposition with PCA. The source of the correlation is the maximum at the gaze location at the time lock which occurs in both PC1 − PC2 and PC1 + PC2 and corresponds to chasee and chaser respectively. While it is theoretically possible that subject 1 focused a group of multiple agents stretched out over the axis of their motion direction, such an interpretation of PC1 is inconsistent with PC2 to PC5, which show two agents.

The agent velocity (*v* in the first row of Table 4) is slightly less than the nominal speed of 14.5. This is probably the case because the agents movement direction did not perfectly correspond to the dominant direction which was used to rotate the samples. As a consequence, the samples did contain a small motion component that is orthogonal to the dominant motion axis. This component was then lost when computing the rail diagrams which discard the orthogonal axis.

## The targets of exploration and catch-up saccades

### Results: principal component analysis

Saccades determine which agents are brought into focus. As such saccades play an important role in the detection process. In this section we present the results of PCA for ES and CS1. CS2 were also analyzed. The results were similar to CS1 and we omit their presentation. Due to the small sample size, it was not worth to analyze the later catch-up saccades (CS3+) separately. Saccade onset was used as time lock and the saccade target was used as positional lock. The time window stretched from 400 ms before the saccade onset to 400 ms after the saccade onset. The positional window was a rectangle of width 10 × 10 degrees with the positional lock at its center. As in the previous section, the samples had been rotated such that they were aligned with respect to the dominant motion direction.

**Figure 5:**
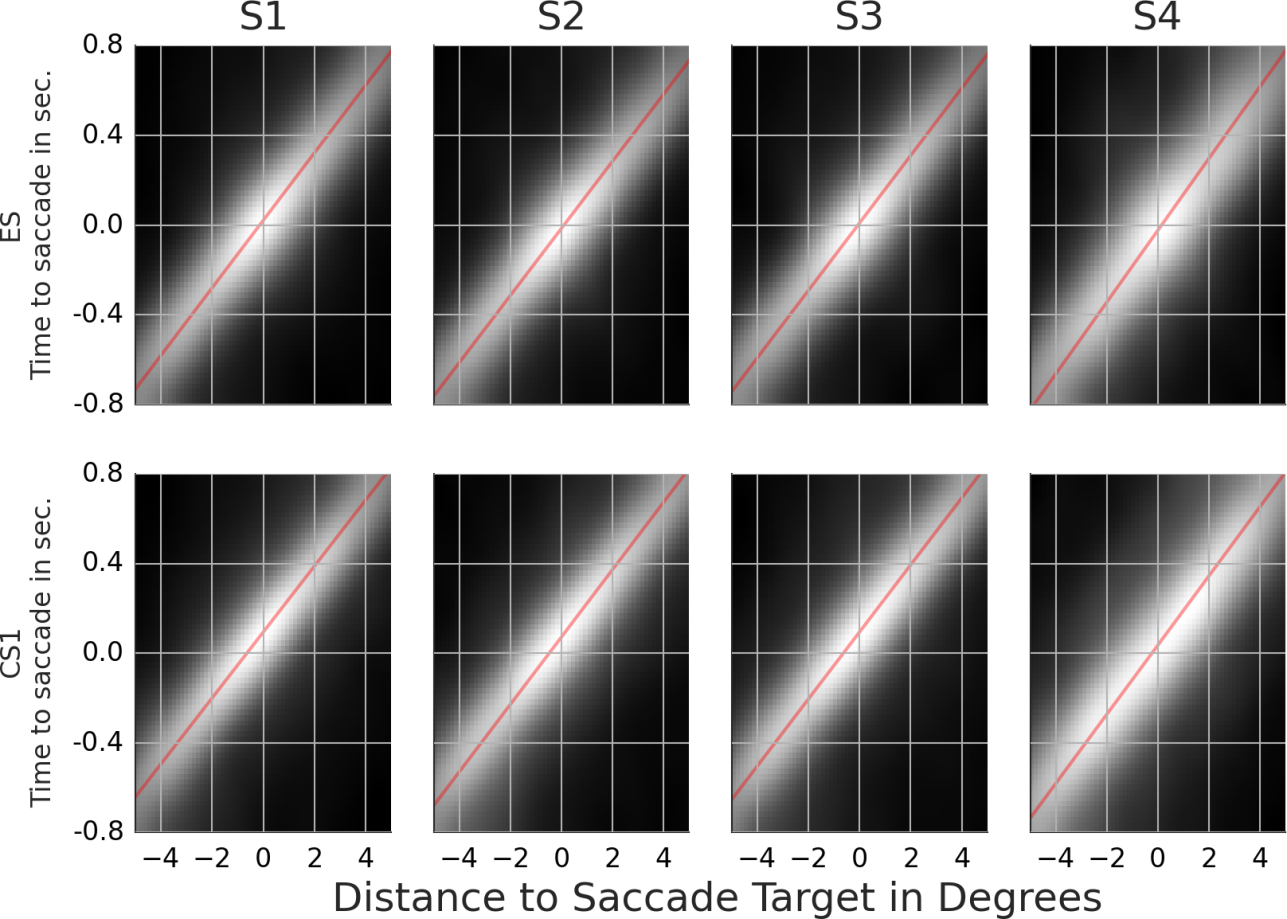
Rail diagrams of the first principal component of the saccade targets. Each column shows templates for one subject. First rows shows results for ES. Second row shows results for CS1. The vertical axis shows time with respect to saccade onset in seconds. The horizontal axis shows the distance from the saccade target in degrees. Agent’s motion manifests as an elongated maximum where the slope along the elongation can be interpreted as the agent’s velocity. To estimate the mean velocity, lines (red) were fitted to the maxima.

Movie S5 shows the first ten principal components for each subject, ordered according to the proportion of the explained variance. The second row of Table 5 shows the proportion of variance explained by the first five PCs in Movie S5. The first row in Fig. 5 shows the rail diagrams of PC1 for each subject and the corresponding parameter estimates are listed in Table 4 ES and CS1. The components are very similar across the subjects. The first component shows a single circular maximum moving across the field. The second and third PC, respectively, shift the maximum of PC1 in horizontal or vertical direction by approximately 0.5 degrees. The maximum in PC1 can be shifted with arbitrary precision by additional PCs with a higher number of smaller peaks and pits (such as PC4 and PC6 of subject 1). PC4 and PC6 differ between subject 1 and the remaining subjects. PC4 and PC6 of subject 1 show horizontal and vertical ternary shift patterns. Subjects 2, 3 and 4 show a different decomposition. PC4 shows a clover pattern, while PC6 resembles a donut. Note however that by adding or subtracting the clover and donut patterns one obtains the ternary shift patterns of subject 1. PC4 explains 1.5 to 1.6% of variance while PC6 explains 1.4%. As a consequence, the choice between the clover-donut decomposition and the shift decomposition relies on less than 0.2% (1.6 − 1.4) of variance and is thus hardly worth considering. The remaining PCs show more complex patterns where the pattern is shifted both, horizontally and vertically (e.g., PC5), or where the vertical position of the pattern changes between before and after the time lock (e.g., PC9).

CS1 were analyzed in the same manner. The PCs are shown in Movie S6. Rail diagrams of PC1 are shown in the second row of Fig. 5 and the parameter estimates are listed in Table 4. PCs of CS1 are with few exceptions qualitatively identical to the PCs of the exploration saccades. For CS1 the clover-donut decomposition is missing in all subjects. As shown in the third row of Table 5, all of the listed CS1 PCs, but especially PC1, explain a larger proportion of variance, indicating a more consistent saccade programming of CS1 over ES.

Next, we compare the results of line fitting between ES and CS1. The speed of the maximum of subjects 1, 2 and 3 is similar and ranges from 13.3 to 13.6 degrees per second. The target of exploration saccades corresponds to the position of the maximum at the saccade onset. The target of CS1 is located ca. 0.5 degrees in front of gaze on the dominant motion axis. Another way to look at this difference is to compute *p*_CS1_/*v*_CS1_ − *p*_ES_/*v*_ES_ which gives the time difference between when the maximum crossed the saccade target of CS1 compared to ES. For CS1 the saccade target was crossed by the maximum 36, 44, 43 and 30 ms later than for ES. In sum, the CS target locations where the maximum will be located ca. 40 ms after the saccade onset. Finally, note that the maximum in the rail diagram of subject 4 is considerably wider (compare *s*_1_) and slower than that of the other subjects.

### Results: classification movies for contrast between the exploration saccade and the first catch-up saccade

In the previous section we already made some tentative comparisons between the PCs of ES and CS1 events. In this section we make the comparison more direct by computing the classification movies between ES and CS1, which should tell us what patterns trigger pursuit.

**Figure 6:**
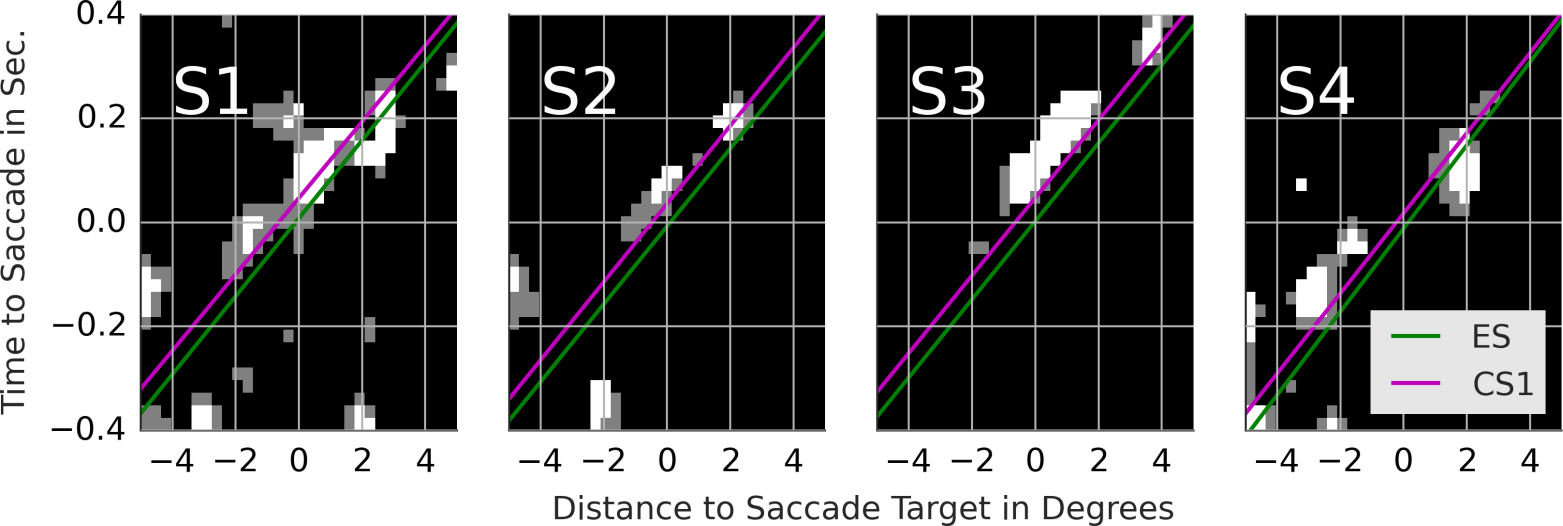
Rail diagrams of the classification movies. Each column shows templates for one subject. The vertical axis shows time with respect to saccade onset in seconds. The horizontal axis shows the distance from the saccade target in degrees. The green and purple lines show the lines fitted to the maxima of ES and CS1 in Fig. 5.

As in Kienzle et al. (2009) we use a support vector machine (SVM) to obtain the classification movies. The details pertaining to the SVM training are presented in section 1 of Supplemental Information 1. We determined the classifier performance with cross-validation. For subjects 1–4, the SVM classified 76.0, 71.3, 74.4 and 76.9% of the samples correctly. The corresponding expected chance performance was 75.0, 69.9, 73.5 and 75.5% respectively. Of most interest to us are the classification images. We followed Kienzle et al. (2009) and determined the maxima and minima of the SVM’s objective function. The classification movies for each subject are presented in the left-most column of Movie S7 (The remaining columns are discussed in the supplement). The maxima show the motion of a single point. The minima show noise. There is no trace of a chasing pattern. Figure 6 shows the rail diagrams of the classification images in the left-most column of Movie S7. The colored lines correspond to the red lines from Fig. 5. The classification maximum of subjects 1, 2 and 3 intersects with the template maximum of CS1. In the case of subject 4, the classification maximum first trails the template maximum of CS1, but after the saccade onset it trails the template maximum of ES.

### Discussion

With 1–1.5% points above the chance level, the SVM performs poorly. Note that the lack of coder reliability (see fourth row in Table 2) can’t explain the poor classification performance. With an overlap of 89%, there is enough space for a performance improvement beyond 71–77%. The relatively poor classification performance of the SVM shows that there are no notable differences between the ES and CS1. Additional analyses show that the objective function of the trained classifier has a single maximum, which likely accounts for the 1–1.5 performance points above the chance level. This maximum can be more easily interpreted by comparing the classification maximum with the template maxima of ES and CS1. With subjects 1, 2 and 3, the CS1 maximum crosses the saccade target location ca. 40 ms after the saccade onset rather than concurrent with the saccade onset as is the case for ES (consider *p*_1_/*v*_1_). SVM exploits this difference as the classification images show a maximum that trails the CS1 template maximum. With subject 4 the main difference concerns the velocity *v*_1_ of ES and CS1 template maxima. Samples with a maximum’s offset of ca. 0.5 degrees before the saccade onset and no offset after the saccade onset are classified as CS1.

Parts of the maxima in the classification movies and in the corresponding rail diagrams are missing. To simplify the derivation of maxima and minima, we thresholded the classification movies. The classification movies thus contain only black and white pixel values (The grey pixels in the rail diagrams are a product of averaging of the center-most pixel rows.). As such, some parts of the movement may be missing, because the signal was weaker and did not exceed the threshold. On the other hand, it is possible that the maxima of classification movies are rather noisy, which would be expected considering the poor classification performance.

The saccade maxima are considerably wider than the maxima we observed with detection events (*s* in Table 4). Hence, we prefer to interpret the PC1 of ES and CS1 as targeting regions with a high density of agents. Such saccade behavior fits with the predictions of the model of bottom-up saliency by Itti & Koch (2000). The locations with high agent density correspond to locations with high contrast saliency. Kienzle et al. (2009) provided an alternative formulation of the saliency model. By adding PC1 and PC6 in Movie S5, one can obtain the center–surround patterns in the top row of figure 3 in Kienzle et al. (2009). Furthermore, since the agents were constantly moving, the locations with high agent density correspond to locations with high motion saliency (Itti & Baldi, 2009).

While both, ES and CS1 target individual agents, two notable differences emerged from our comparison of ES and CS1. First, PC1 of CS1 were more precise than PC1 of ES as evidenced by the higher proportion of explained variance. Second, the PCA results indicate that the CS1 maximum crossed the saccade target location ca. 40 ms after the saccade onset rather than during the saccade onset as was the case with ES. The importance of this difference between ES and CS1 was also supported by the analysis of classification movies. ES were directed to the location of high agent density at the saccade onset. CS1 were directed to where the density maximum will be when the saccade is half-way finished. In conclusion, the CS1 programming involved a prediction mechanism while ES programming did not. Both, the higher precision of catch-up saccades as well as their predictive nature have been documented in the studies of smooth pursuit eye movements (Lisberger, 1998; De Brouwer et al., 2002; Barnes, 2008). The higher precision and the predictive nature reflect the choice of the event identification procedure. As such, these results highlight a good accuracy and validity of this procedure.

In the case of subject 4, the SVM classifier exploited the velocity (*v*) difference between ES and CS1. Since all agents moved with the same velocity, we think that the velocity difference arises through worse precision of the catch-up saccades of subject 4 as evidenced by higher *s* value. Less precise saccades lead to a higher proportion of velocity being lost by discarding the dimension orthogonal to dominant movement direction. In fact, the 8 pairs of *s* and *v* of ES and CS in Table 4 correlate with *r* = 0.95. The worse precision of the saccades of subject 4 can be either a consequence of worse visual acuity or (in our opinion more likely) of worse eyetracking data quality.

In conclusion, we did not discover any particular patterns that trigger pursuit. Rather, the subjects pursue groups of multiple agents and these are further judged during smooth eye movement. The saccades help to bring candidate agents into the focus, but do not contribute to the detection of chase beyond that. As a consequence the crucial evidence must be gathered during smooth eye movement, which we look at in the next section.

## Smooth eye movement

### Results

Smooth eye movement aligns the gaze with a moving object such that the object appears stationary on the retina, which provides opportunity for precise inspection of the object and its features. In this section we look at what happens between two consecutive catch-up saccades. We refer to the period between two consecutive catch-up saccades as a smooth movement episode. Since smooth eye movement has an orientation, the samples were aligned with respect to the gaze direction rather than with dominant motion direction. Another advantage was that, with smooth movement episodes, we had identified the pursued agents during event identification. This allowed us to remove the remaining agents when constructing the samples and so to improve the signal-to-noise ratio of the template movies. A disadvantage is that, unlike saccade or detection events, the length of smooth movement episodes varies. As a consequence, it was not possible to perform PCA. Even when relying on averaging, we had to decide whether we put the time lock at the start or at the end of the episode. As another difficulty, the number of smooth movement episodes is high and the episodes can be longer than a second. To speed up computation we used non-parametric regression to compute the average template.

**Figure 7:**
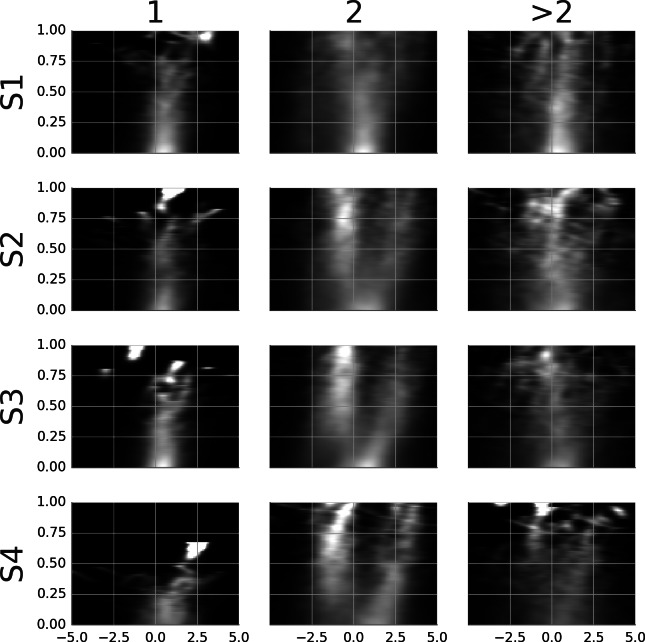
Rail diagrams of the average templates of smooth movement episodes with time lock at the start. Each row shows average templates for a different subject. The columns show templates for samples with one, two or more than two agents. The vertical axis shows time with respect to the time lock in milliseconds. The horizontal axis shows distance in degrees from the positional lock on the horizontal axis of the corresponding panel in Movie S8.

Movie S8 shows the average template of samples locked at the start of the smooth movement episode. The positional lock is at the gaze location at the respective time point. Thus, the templates show stimuli with subtracted smooth eye movement, i.e., how they would appear on the retina during smooth eye movement. The average templates were computed separately for samples with one, two, and more than two pursued agents. These are shown in the columns of Movie S8. The proportion of episodes with one agent was 23.9, 18.7, 15.2 and 9.4% while the proportion of episodes with two agents was 66.8, 61.5, 69.5 and 71.1%. Figure 7 shows the rail diagrams corresponding to the template in Movie S8. The rows show four subjects. The first column shows a single circular maximum located 0–1 degrees in front of the gaze. The third column shows a wide circular maximum—a group of agents, with highest density at the gaze position. The second column shows a single maximum which after 200 ms blends into two distinct maxima (subjects 2, 3 and 4), or it stretches horizontally (subject 1). The patterns after 200 ms are similar to those obtained in the analysis of the detection events. Note that the number of samples that contribute to the average gradually decreases. As a consequence, the agents from the individual samples become discernible towards the end. In addition, the samples may be misaligned towards the end which adds to the noise in the template movies.

**Figure 8:**
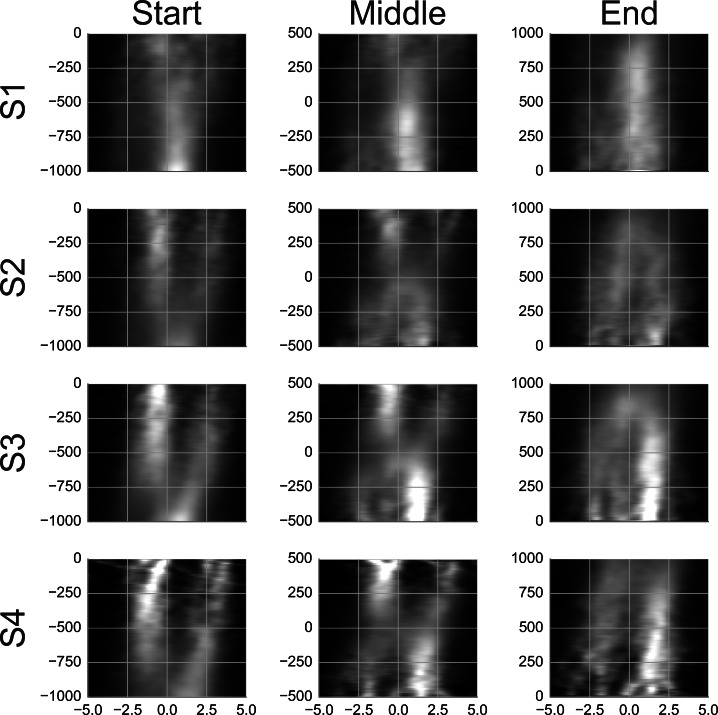
Rail diagrams of the average templates of smooth movement episodes with two agents. Each row shows average templates for a different subject. The columns show templates locked at the start, in the middle and at the end of the smooth movement episode. The vertical axis shows time with respect to the time lock in milliseconds. The horizontal axis shows distance in degrees from the positional lock on the horizontal axis of the corresponding panel in Movie S9.

To see what happens at the end of a smooth movement episode, the third column of Movie S9 and Fig. 8 display the average templates with a time lock at the end of the episode. Movie S9 and Fig. 8 show only the samples with two agents. These were the most common and are of most interest to the present study. To facilitate a comparison, the first column in Movie S9 and Fig. 8 reproduces the second column from Movie S8 and Fig. 7. Looking at the end-locked templates, subjects 2–4 show two maxima. The template of subject 1 shows one horizontally stretched maximum located 0–1 degrees in front of the gaze point. The patterns blend to a single maximum during the last 200 ms.

**Figure 9:**
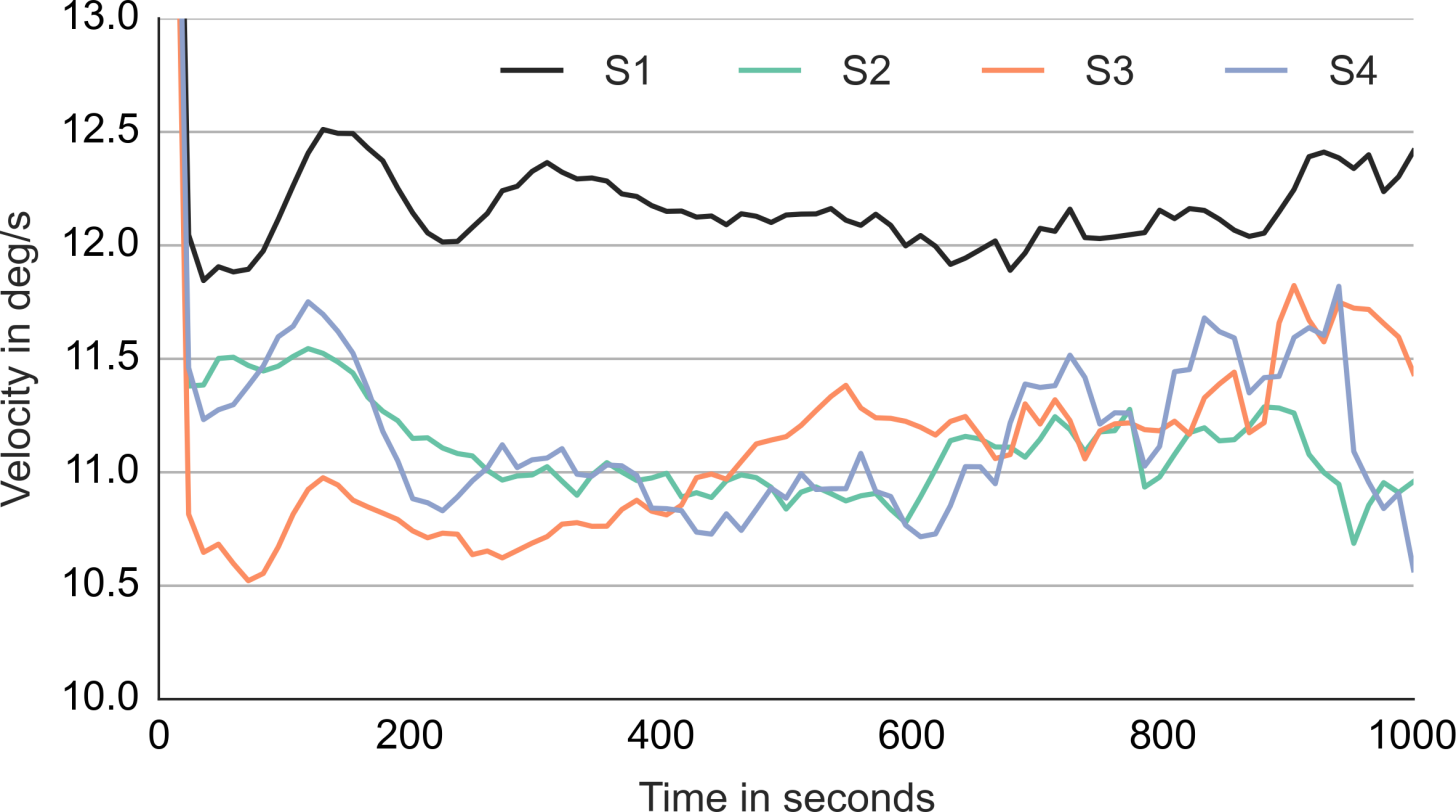
Median gaze velocity during smooth movement episodes in degrees per second. The horizontal axis shows the time since the start of the episode in milliseconds.

Comparing the first and the third column, the maximum in front of the gaze is initially stronger while the maximum behind the gaze takes over toward the end. To get a better idea of what happens in between, a template with samples aligned in the middle of the episode was computed and is shown in the middle column of Movie S9. Just like the start-locked and end-locked templates, the mid-locked templates indicates a switch between the two maxima. The switch is achieved by setting the smooth movement velocity approximately 2 degrees slower than the agent velocity and by locking the smooth movement to the chaser when he comes into the vicinity of gaze. Indeed, Fig. 9 shows that the median gaze velocity during smooth movement episodes of subjects 2, 3 and 4 is one degree slower than that of subject 1.

### Discussion

The templates of smooth movement episodes are similar to those observed at detection. This is not surprising as at detection the subject would engage in smooth eye movement. The templates of subject 2–4 indicate a switch between two agents. An instance of this switching strategy can be observed in Movie S1 which shows a trial from subject 3. The existence of such agent switch is further supported by the fact that the median gaze velocity of subjects 2, 3 and 4 (Fig. 9) is 3–4 degrees slower than the agent velocity (14.5 degrees). The gain of smooth eye movement is usually between 0.9 and 1 (Meyer, Lasker & Robinson, 1985). This is higher than the 0.75 observed in the current case. Steinman, Skavenski & Sansbury (1969) demonstrated that people can voluntarily decrease the smooth movement velocity and pull the gain below 0.9. We think that the small gain observed in the current study reflects a unique pursuit strategy that works well with chasing motion. If the catch-up saccade is directed to the chasee, the chaser usually follows the chasee with ca. 3.5 degrees. The observer is able to switch the gaze between the two agents within a second without making a saccade. Such pursuit strategy provides another demonstration of top–down control of pursuit eye movements (Barnes, 2008).

We think that the templates of subject 1 indicate a similar behavior as she has shown at detection. Subject 1 prefers to track individual agents. Subject 1 makes consecutive saccades between agents in the group. Movie S10 shows a trial from subject 1 that illustrates her tracking strategy. This can be compared with Movie S1 which shows a trial from subject 3 and illustrates the group-tracking strategy. Unfortunately, we could not perform PCA with the samples from smooth movement episodes. Nevertheless, we think that the similarity of the PC1 template of detection events (Movie S3 and Fig. 4) to the average template of smooth movement episodes (Movie S9 and Fig. 8) along with the subject-wise comparison of median velocity (Fig. 9) make such interpretation plausible. Furthermore, the pursuit of individual agents is the more conservative strategy. It allows more accurate inspection of the agent’s motion but the inspection of multiple agents requires more time since the subject needs to repeatedly switch between the agents. The more accurate but slower responses of subject 1 then fit with her preference for the conservative pursuit strategy.

Movies S1 and S10 indicate that the different tracking strategies are a matter of degree. Subjects 2–4 switch to pursuit of individual agents if these came further apart, or if some wild motion change makes it difficult to track the group as a whole. Subject 1 switches to group tracking if the two agents come close together. This would be mostly the case in the NC trials.

The templates of subjects 2, 3 and 4 in Movie S9 and Fig. 8 show single agent during the first and last ca. 200 ms. Furthermore, a non-negligible proportion of episodes of these subjects focused single agent. Don’t these observations contradict our claim that subjects 2, 3 and 4 prefer to track two agents as a group? Let us ask how such single agent samples (irrespective of whether they were coded as showing one or two agents) arise. First, they may arise through misclassification of exploration saccades (which would be followed by fixations rather than by smooth eye movement) with catch-up saccades during event identification. As Table 2 shows (CS sensitivity), this may account for up to 14% of the cases. Second, as already mentioned, subjects 2, 3 and 4 apply the strategy of subject 1 at least to some degree. This would result in samples that focus on a single agent. Third, when templates with chase patterns show a switch from the chasee to the chaser, the chasee maximum in the rail diagrams (Fig. 8) is stronger at the start, while chaser is stronger at the end. The strength of the single agent increases up to a degree at which the second agent vanishes. Thus, the single agent pattern at the start and the end is not necessary inconsistent with the switch pattern. Finally, one may consider the single agent samples as failed two agent samples. If the chaser changes its motion direction, a corrective catch-up saccade may be necessary to keep both agents in view. Probably each of these explanations has some merit. It is possible to further investigate their contribution empirically with the current data, though we refrain from doing so as this would imply a departure from the current approach that focuses on template analyses. A conclusion from the third and the fourth consideration is that one should conceive of the agent switching strategy as the preferred rather than the most common tracking strategy.

Meyerhoff, Schwan & Huff (2014b) provided evidence that attention is dedicated to a single agent at a time, rather than to a pair. In experiment 4, they showed that it is easier to find the chaser when the chasee’s identity is given than vice versa. They argued that an equal distribution of attention implies similar performance in both cases. In experiment 5, they showed that the detection time increases linearly with the number of distractors. In contrast, the number of pairs increases quadratically with the number of agents. At first look, their finding conflicts with the claim that subjects 2, 3 and 4 pursue chase as a group. Note that Meyerhoff, Schwan & Huff (2014b) investigated the allocation of attention while the current study investigated the gaze location. Heinen, Jin & Watamaniuk (2011) have demonstrated that attention can be disengaged from the object at the center of pursuit. Furthermore, Watamaniuk & Heinen (2014), demonstrated that the allocation of attention during pursuit is broad and flexible. Then, the gaze location and its alignment with respect to the agents provide only a weak information about the allocation of attention among the tracked agents.

Pratt et al. (2010) demonstrated that attention is drawn more strongly by agents that show eccentric rather than smooth motion. In contrast, the average templates in Movie S9 and Fig. 8 show smooth movement. Note that in Pratt et al. (2010) the subjects made no pursuit eye movements. The subjects were asked to fixate a point and to broadly allocate their attention. Hence, their findings are not comparable with the findings in the current section. In as far as allocation of attention precedes a saccade (Kowler et al., 1995), the findings by Pratt et al. (2010) can be contrasted with the findings for ES in the current study. PC9 and PC10 templates describe direction changes. However, the proportion of variance explained by these components is low. The current study thus does not indicate that the subjects’ saccades and attention are attracted by direction changes. Note that the search context in Pratt et al. (2010) was different. In Pratt et al. (2010) the distractors were inanimate, while in the current study all agents were animate. These task differences may account for the different findings.

Fehd & Seiffert (2008) and Fehd & Seiffert (2010) investigated the strategies used by people when pursuing multiple objects. Fehd & Seiffert (2008) showed that people use two strategies: they either pursue the agents by shifting the gaze between individual agents, or they pursue the agents as a group. As a part of the latter strategy, people align their gaze with the center of the group. We observed similar two strategies in the current study. However, in the current case, the group tracking strategy showed low gain of ca. 0.7 and should be considered as distinct from a pure centroid tracking strategy in Fehd & Seiffert (2008). Unfortunately, Fehd and Seiffert did not investigate the properties of catch-up saccades and smooth eye movements (and their gain in particular) and as a consequence, it’s difficult to compare the group tracking strategies.

## Ideal Observer

### Results

In the introduction, we mentioned that one way to check whether the template images reflect features of cognition rather than the experimenter’s stimulus choice is to ask whether the subjects behave differently than the ideal observer. In this section, we look at the templates from samples that are centered on the chasing pair. The identity of the chasing pair was given by the trajectory generation algorithm. A total of 5,000 samples were extracted from the trajectory data that were shown to the subjects during experiment. The template analysis was identical to that used with the saccade samples. In particular, the samples were aligned with respect to their dominant motion. The time lock was chosen randomly such that the start and the end of the time window was located inside a trial. The time window went from 400 ms before to 400 ms after the time lock. The sample window was set halfway between the chaser’s and the chasee’s position at time lock. In this analysis we were interested in the agents’ relative motion pattern, not in how the pattern aligns with the gaze point. As such, the precise choice of the positional lock is irrelevant as long as it consistently focuses the chasing pair.

**Figure 10:**
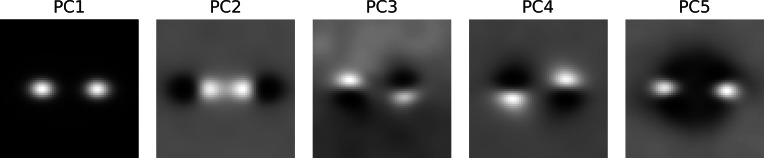
Principal components of the ideal observer. Each cell shows a snapshot from Movie S11 at the random time lock.

The first five PCs with most explained variance are shown in Movie S11. A snapshot in Fig. 10 shows each PC at the random time lock. The first PC in the first panel from left shows chase. We obtained estimates of the velocity and of the distance between the two agents by fitting lines to the rail diagram of PC1 (not shown). The chasee and the chaser were 3.43 degrees apart and traveled 13.99 and 13.93 degrees per second (Table 4 IO). The PC2 contributes to samples with different chaser-chasee distance. The chase pattern in the NC trials scores high on PC2. The chase from the DC trials scores low on PC2. The remaining three PCs show different kinds of oblique motion. Due to the minimum distance constraint in DC trials, the chasing pair tends to run in circles and the remaining PCs can be summed to construct this kind of motion. Whether the circling motion is clockwise or counter-clockwise is determined by the sign of the PC. The variance explained by the five PCs in Movie S11 is listed in Table 5. Even with perfect positional alignment, the first PC achieved only 16.8 percent of variance.

**Table 5:** Proportion of explained variance for the first five highest scoring principal components. The range in each cell gives the minimum and maximum value across the four subjects.

Event	PC1	PC2	PC3	PC4	PC5
Detection	[6.8, 8.0]	[3.3, 3.8]	[2.4, 2.8]	[1.9, 2.1]	[1.8, 2.0]
Exploration saccade	[3.7, 4.6]	[2.3, 2.4]	[1.9, 2.0]	[1.5, 1.6]	[1.4, 1.5]
Catch-up saccade 1	[6.0, 7.6]	[2.5, 2.9]	[2.0, 2.1]	[1.7, 1.9]	[1.5, 1.8]
Ideal observer	16.8	3.4	2.1	1.8	1.7

### Discussion

Let us compare the PC1 values across the rows of Table 5. The proportion of variance explained by the PC1 (the chase pattern) of the ideal observer was higher than that of any of the subjects’ events. As expected, the ideal observer was more precise and efficient at targeting the chase than the human subjects. Even though the subjects were less precise, a comparison between Movies 11 and S3 and the rows BT and IO in Table 4 shows that the subjects targeted the same motion pattern as an ideal observer. The distance between the two agents was similar. The velocity of the subjects’ chase pattern was slightly lower, probably due to the less exact orientation alignment. On the other hand, we did not observe PC2, PC3 and PC4 of ideal observer with human subjects. This means that subjects did not exploit the oblique motion as a cue to chase. At the same time, the subjects seem to have discarded the evidence from the NC trials and they searched for two agents moving 3.5 degrees apart. This is also suggested by the worse accuracy and longer response times in NC trials (Fig. 2).

Do the templates reflect aspects of the human cognition or rather the experimenter’s stimulus choice? The results in the current section show that the second possibility should be seriously considered. We turn to these considerations in the next section.

## General Discussion

The main contribution of the current study is to decompose the chase detection behavior of subjects into elementary gaze events and to separately describe the behavior during these events. The results highlight the importance of pursuit and smooth eye movements for the detection of chasing motion. We discuss their importance in the next subsection. The goal of the current study was however more ambitious—namely to identify with the help of template analyses the motion patterns that are used by subjects during these events to detect chase. While the template analyses provided interesting results, their interpretation is hampered by the evidence of learning (Fig. 3) and by the fact that subjects’ behavior was similar to that of the ideal observer. This issue is discussed in the second subsection. The third subsection discusses the validity of the rather uncommon methodology used in the current study. We discuss the choice of methods for template analyses. To our knowledge, this is the first study that attempts to identify pursuit eye movements in a task that allows both, fixations and smooth eye movements. The validity of the event identification procedure is discussed in the third section as well. The current study describes the search behavior of subjects in sufficient detail to provide a basis for a computational model of the chase detection process. We discuss how such model may look like in the final section.

### When pursuit tracks chase

The template analyses showed that pursuit and smooth eye movements in particular help to stabilize on the retina a pattern that consists of two agents moving one after another in the same direction. Does this mean that such motion pattern represents the positive evidence on which the detection decision is based? The analyses of pursuit episodes (Movie S9) and detection events (Movie S3) can’t be considered as a full support for such thesis. As already discussed, attention is another factor that may constrain an observer’s judgment further (Meyerhoff, Schwan & Huff, 2014b).

While smooth eye movement may not fully determine which features are considered as evidence of chasing, smooth eye movements determine which features are *not* considered. During pursuit, the features in the background are difficult to attend to (Khurana & Kowler, 1987; Schütz et al., 2007) and pursuit can be considered as a selective filter that, similar to attention, helps to select agents for further inspection. As a consequence, people with degraded smooth pursuit abilities, such as infants (Aslin, 1981; Von Hofsten & Rosander, 1997; Pieh, Proudlock & Gottlob, 2012), autists (Takarae et al., 2004) or seniors (Zackon & Sharpe, 1987) may result in different eye movement behavior and impair the detection of goal-directed motion. With chase in particular, pursuit eye movements may enhance chase detection beyond the typical gains provided by pursuit during tracking of moving stimuli (e.g., Schütz et al., 2008). Pursuit is a type of chasing motion and hence it is plausible that the motion characterics of pursuit eye movements and chasing motion will overlap. As a consequence, the subjects may engage in long pursuit episodes in order to improve detection accuracy. Without any need to actually inspect the pursued agents, the mere fact that a pursuit of a group of agents has been sustained for a long period can be used as a criterion for chase detection. Such a detection strategy is interesting because it may help explain how chase and social motion in general is subjectively perceived. Scholl & Gao (2013) pointed out that “the observers simply see animacy and intentionality when viewing the displays, effortlessly and automatically, and without any instructions or preparation. “The chase detection may feel automatic and effortless because the pursuit eye movements that accompany it feel automatic and effortless. Such a hypothesis can be tested by comparing the reports of subjective experience and the performance when pursuit eye movements are allowed, avoided or impaired.

### Learning and feedback

The behavioral results suggest that subjects do not bring a preformed template to the task. Rather, subjects bring certain hypotheses about what may qualify as a chase and then, through feedback, they converge to a single pattern. We have observed the convergence process in Fig. 3. Note that the unfamiliarity with the task and the controls may account for one or two mistakes at the start of the experiment, but can’t explain the poor performance. We have also shown that subjects converge to a solution favored by the ideal observer and by the feedback.

Can we get a glance at the subject’s hypotheses about chasing motion were before they adjusted their response to the feedback? We looked at the early trials where the subjects made mistakes. Subject 3 made 12 false positives in her first 25 trials—the most of all subjects. Her learning history is thus quite instructive. Agents in the current experiment moved all with equal speed. Nevertheless, it was possible to observe an approach when the chasee moved perpendicular with respect to the chaser’s direction. Movie S12 shows an early trial from subject 3 in which she reported detection after observing an approach. Another early trial in Movie S13 shows two agents moving almost in parallel. These were also flagged as chase. The subject tried out a wide spectrum of hypotheses about chase-like motion and she utilized the task feedback select one that maximized her performance.

The presence of rapid reinforcement learning is troubling because, as we discussed in the introduction, the results of the template analyses are then likely constrained by experimenter’s stimulus choice. This does not make the findings invalid but it limits their generality. If the experimenter chose a wider range of stimuli subject may have shown different behavior. Consider for instance the two pursuit strategies of subject 1 and of the remaining subjects. Had we chosen agents that move smoothly and close together, the subjects may have focused groups of agents instead of individual agents. Had we chosen agents with a more jittery motion, the subjects may have pursued individual agents more often. Furthermore, had we selected smaller, slower, and fewer agents, pursuit eye movements might not be as important and a single saccade to the target region might have sufficed to identify the chase. The reader should keep these considerations in mind when comparing the results of studies of goal-directed motion, that use different tasks and stimuli.

Note that the omission of feedback does not resolve the issue. Even without feedback, the subject may infer some criterion based on the distribution of the motion features and behave in accord with this criterion. (Or, worse, the subject will show non-stationary behavior, making any evaluation of the data difficult.) With respect to future research, if the observed behavior depends on the experimenter’s task and stimulus choice, then we suggest that researcher should ask what the most relevant tasks and stimuli are. One option is to increase the variety and scope of stimuli and tasks used to study perception of goal-directed motion. Another promising option is to focus on stimuli and tasks that match natural contexts where chasing arises. In our opinion, chasing games in kindergarten children (Pellegrini et al., 2004), goal-directed action in sports (Taya, Windridge & Osman, 2013), and computer gaming are good candidates.

### The choice of event identification procedure and other decisions

In this section we discuss the analysis-related decisions and whether these compromise the validity of the presented results and conclusions. First we discuss the validity of the procedure for identification of complex events. The features and properties of pursuit eye movement that have been reported in the literature are also found with events that were coded as pursuit in the current study. This shows that our operationalization of exploration and pursuit and subsequent event identification were successful. One example of such convergence are the velocity oscillations of smooth eye movements. These oscillations are apparent in Fig. 9 during the first 200 ms. Similar oscillations have been described in the literature (Robinson, 1965). To give another example Barnes (2008), documented the involvement of prediction mechanisms in pursuit eye movements. In the current study, catch-up saccades were, in contrast to exploration saccades, predictive. One not expect such prediction from exploration saccades.

Another way to consider the validity is to ask how a different operationalization of the event identification would alter the results. First, note that the analysis of the detections did not utilize any information about complex events. Second, as an instance of an alternative operationalization consider what would happen if we had skipped the manual correction and used the results of the automated identification. As already noted, the automated procedure incorrectly split pursuit when the tracked agents moved farther apart. Apart from separating the pursuit events such a mistake would also flag the saccades when the agents were farther apart as ES instead of CS. With smooth movement episodes, this would actually work in favor of confirming the obtained templates, since all the cases where the agents came farther apart but where the subject nevertheless kept tracking them—all these cases would be flagged as exploration.

While the pursuit identification procedure used in the current study is laborious and complicated, we currently don’t see any better alternative. One popular strategy is to digest raw eye movements with an algorithm that extracts only fixations and saccades (e.g., Klein et al., 2009; Roux, Passerieux & Ramus, 2013). Episodes of smooth eye movement are then decomposed into fixations. The eye movement accuracy is computed not against the actual gaze position but against the fixation target which is an average of the moving gaze position throughout the fixation. This procedure may introduce noise such that actual differences in accuracy between groups can’t be discerned anymore. This procedure can also lead to biased results when groups and/or conditions differ in how pursuit eye movements are deployed. The current study is to our knowledge the first study that attempts to identify pursuit eye movements in a competitive task that requires both exploration and pursuit. As such we see the manual procedure as a first important step towards a fully automated pursuit identification algorithm.

We also made multitude of decisions with respect to methods that were used to analyze the data. For instance, we used two different methods to align the rotation of the samples and we used two methods to derive templates. Note however that many of the analyses overlap. Pursuit episodes start and end with catch-up saccades and the detection events consist almost exclusively of smooth movement. If we compare the results of the overlapping analyses we see that these agree. In addition, the analysis of data from an ideal observer can be considered as a validation procedure. The results of this analysis were in accord with what we know about the stimulus generation procedure. We think that, if anything, the methodological diversity highlights that the results are robust and independent of the data-analytic decisions.

### Towards a computational account of chase detection

We framed the current exploratory study as a first step in a reverse-engineering attempt. This attempt should result in a computational model of human chase detection. Such a model should not only predict detection performance but also eye movements during the search. The model can then be used to derive predictions for stimuli and tasks used by other studies (Gao, Newman & Scholl, 2009; Gao & Scholl, 2011; Meyerhoff, Huff & Schwan, 2013; Meyerhoff, Schwan & Huff, 2014b). The model can tell us how these findings, which all use slightly different stimuli and different tasks, fit together and where they disagree. In the current study we performed no computational modeling nor was any intended. In this section, we offer a brief sketch how such a computational model may look like. This sketch draws on the results of the current study and hence demonstrates its usefulness.

We suggest the following model. Contrast saliency (Itti & Koch, 2000) determines the targets of exploration saccades. If two consecutive saccades target the same global saliency mode, pursuit is initiated with the second saccade (which corresponds to CS1). During the pursuit, evidence is computed as the correlation between the retinal image and the appropriately rotated version of a template with two agents (for instance the one in the left-most panel in Movie S11). The evidence is accumulated across consecutive smooth movement episodes. A two-choice drift diffusion process (Ratcliff & McKoon, 2008) is used to turn the evidence into a decision to terminate the pursuit with a detection or to continue with exploration. Additionally, the diffusion process determines the overall pursuit duration. The thresholds and other parameters of the diffusion process can be derived from the saccade counts and from the detection times. A simple way to determine the duration of individual smooth movement episodes is to draw a random value from an exponential distribution that mimics the distribution of the duration of smooth pursuit episodes observed in the current experiment. The fixation duration during exploration can be determined in a similar manner. Finally, the template can be used to determine which of the two pursued agents is the chasee and in which direction the two agents move. As with subjects 2, 3 and 4 the catch-up saccades target the chasee. The smooth movement follows the agents’ direction. To produce agent switching, its velocity is set to 0.75 of the target velocity.

The above model is rather simple and makes some assumptions that go beyond the current evidence, but precisely for this reason it would be interesting to know whether and how well it can account for the phenomena reported in the literature. The setup and the testing of such computational model is an endeavor for future research. We hope that the above considerations highlight the value of the current study. Ultimately, the researchers who studied the alien device wished to simulate its working with their own models. Their investigation would be accomplished once they could simulate the alien device in sufficient detail and with sufficient accuracy. Once again, we think that the same reasoning applies to the study of the perception of goal-directed motion.

## Supplemental Information

10.7717/peerj.1243/supp-1 Supplemental Information 1 Supplement referenced in the manuscript The supplement describes SVM classification and how classification images were obtained. The supplement describes non-parametric regression that was used to obtain templates for smooth movement episodes. Finally, the supplement provides comparison of the complex events identified by the automated algorithm and by the two human coders.

10.7717/peerj.1243/supp-2 Movie S1 Movie S1 The movie illustrates a pursuit event where the tracked agents are further than 4 degrees apart at time 3–4 s after the trial onset. The layout of the movie is described in the section on pre-processing of eye-tracking data.

10.7717/peerj.1243/supp-3 Movie S2 Movie S2 The shows a typical example of eye movements during the chase detection task. The layout of the movie is described in the section on pre-processing of eye tracking data.

10.7717/peerj.1243/supp-4 Movie S3 Movie S3 Movie S3 shows the first ten principal components ordered according to the variance explained (columns) for each subject (rows) for the targets of the first catch-up saccade.

10.7717/peerj.1243/supp-5 Movie S4 Movie S4 Each row in Movie S4 shows the result (right-most column) of the addition of two principal components (first and second column).

10.7717/peerj.1243/supp-6 Movie S5 Movie S5 Movie S5 shows the first ten principal components for each subject, ordered according to the proportion of explained variance. The principal components describe the exploration saccades.

10.7717/peerj.1243/supp-7 Movie S6 Movie S6 Movie S6 shows the first ten principal components for each subject, ordered according to the proportion of explained variance. The principal components describe the first catch-up saccade.

10.7717/peerj.1243/supp-8 Movie S7 Movie S7 The classification movies for each subject are presented in the left-most column of Movie S7. The remaining columns are discussed in the supplement.

10.7717/peerj.1243/supp-9 Movie S8 Movie S8 Movie S8 shows the average template locked to the start of a smooth movement episode. We computed average templates separately for samples with one, two, and more than two pursued agents. These are shown in the columns. The rows show the four subjects.

10.7717/peerj.1243/supp-10 Movie S9 Movie S9 Movie S9 shows templates for smooth movement episodes that focused two agents. The columns show templates locked at the start, in the middle or at the end of the episode. Subjects are shown in rows.

10.7717/peerj.1243/supp-11 Movie S10 Movie S10 Movie S10 shows a trial from subject 1 that illustrates her tracking strategy. Subject 1 prefers to track individual agents. She makes consecutive saccades between agents in the group. The layout of the movie is described in the section on pre-processing of eye tracking data.

10.7717/peerj.1243/supp-12 Movie S11 Movie S11 Movie S11 shows the first five PCs with most explained variance computed with the samples from the ideal observer.

10.7717/peerj.1243/supp-13 Movie S12 Movie S12 Movie S12 shows an early trial from subject 3 in which she reports detection after observing an approach. The layout of the movie is described in the section on pre-processing of eye tracking data.

10.7717/peerj.1243/supp-14 Movie S13 Movie S13 Movie S13 shows an early trial from subject 3 in which she reports detection after observing two agents moving almost in parallel. The layout of the movie is described in the section on pre-processing of eye tracking data.

## Additional Information and Declarations

### Data Availability

The following information was supplied regarding data availability:
http://github.com/simkovic/Chase
http://github.com/simkovic/Chase/releases/tag/publication.
