# Supplementary material for: Pursuit tracks chase: exploring the role of eye movements in the detection of chasing
Source: PeerJ. 2015 Sep 15;3:e1243. doi: 10.7717/peerj.1243 (PMC4579031; doi:10.7717/peerj.1243)
Supplement: Supplemental Information 1 — The supplement describes SVM classification and how classification images were obtained. The supplement describes non-parametric regression that was used to obtain templates for smooth movement episodes. Finally, the supplement provides comparison of the complex events identified by the automated algorithm and by the two human coders. [file peerj-03-1243-s001.pdf]

Supplement to pursuit tracks chase

Matúš Šimkovic[1] and Birgit Träuble[1]

[1]**Universität zu Köln, Germany**

ABSTRACT

Keywords:

1 CLASSIFICATION WITH SUPPORT VECTOR MACHINES

We use a support vector machine (SVM) to obtain a classifier that best discriminates ES and CS1. SVM requires a similarity matrix S as input. This matrix describes the pair-wise distance between all samples. In our case, the samples are the ES and CS1 targets x_n. We compute similarity euclidean distance between two samples $S(x_m, x_n) = \sum_f \sum_i \sum_j (x_m(f,i,j) - x_n(f,i,j))^2$ where $x(f,i,j)$ is the value of a pixel at the i-th position from left, j-th position from top of the window and at the f-th frame in the sequence. SVM minimizes $F(w,b) = \sum_m [1 - y_m(\sum_n w_n \cdot K(x_m, x_n) + b)]_+ + \frac{\gamma}{2}|w|^2$. Function $[\cdot]_+$ is $[x]_+ = x$ for $x > 0$ and $[x]_+ = 0$ for $x < 0$. $K(a,b)$ is the Gaussian function $K(a,b) = \exp\left(\frac{S(a,b)}{\beta}\right)$. The variables β and γ are free parameters. β is the precision of the Gaussian function. For large β the similarity becomes $K(a,b) = 1$ for all a and b and the distance between two samples does not play any role. For smaller β the similarity increases more severely with constant distance increments. γ determines the complexity of the classifier (e.g. the number of support vectors). Small γ means a complex model. We use grid search and cross validation to determine β and γ that maximize the classification rate.
Table 1 euclidean distance between two samples shows the results of classifier training. The table also

SID	CL	CH	#ES	#CS1	#SV	%SV	log(γ)	log(β)
1	75.98	75.03	10760	3580	12863	89.70	1.0	0.0
2	71.29	69.88	8272	3566	11640	98.33	0.5	1.0
3	74.44	73.52	11859	4271	15977	99.05	0.5	1.5
4	76.85	75.51	7606	2467	8876	88.12	0.5	0.5

Table 1. Results of SVM training. SID - Subject, CL - classification performance, CH - chance performance CH=#ES/(#ES+#CS1), #ES - number of ES samples, #CS1 - number of CS1 samples, #SV - number of support vectors %SV - percentage of support vectors from the maximum number of support vectors %SV= #SV/(#ES+#CS1)

shows the optimal values obtained for β and γ. By comparing the classification performance to the chance performance it can be seen that SVM performs poorly. The performance is 1-1.5 percentage point above the chance level. In addition, we did obtain non-sparse solutions that use most of the available samples as support vectors.
We look at motion patterns that are used by the SVM to discriminate the two samples. We optimize function F but this time with respect to x while w, b, γ and δ are treated as constants. High F score indicates a CS1 event. In our case x is a vector with $64 \times 64 \times 68 = 278528$ elements (pixel values). To simplify optimization we optimize a $32 \times 32 \times 34$ vector which is then up-scaled to $64 \times 64 \times 68$. Furthermore, we use binary values (on/off pixels) instead of a continuous values. We use simple hillclimbing search to find the maximum and minimum score of F with respect to x. First we generate thousand random vectors (with 10% bright pixels). We select the vector with the highest/lowest score. The selected vector is then further improved. One after another, in random order, we flip each pixel. If a flip results in a more extreme score we keep this change. Otherwise we discard it. We terminate the search when no pixel flip improves F. The search algorithm will always terminate, though it doesn't warrant that we will find the

global optimum of $F(x)$. To see whether there are multiple local optima, we replicate the above analysis four times for different random seeds. All replications showed similar, in most cases identical, results. The obtained optima are presented in the familiar movie format. Movie 8 shows the minimum and maximum for each subject. Subjects are shown in rows. Replications are shown in columns. The background of maxima is black. The maxima show the motion of a single point. The minima show noise. There is no trace of chasing pattern. As the replications are identical and the minima show only background noise, the classification performance can be summed up by any of the maxima. We focus on the left-most column in text.

Finally, we mention some caveats to our interpretation of the SVM results. In the report we claim based on the SVM results that there are no notable differences between the ES and the CS1 events. With negative results (no difference between ES and CS1) there are always multiple reasons why the analysis may have failed. In particular, if we failed to align the samples correctly with respect to their rotation, this would make the classification problem impossibly difficult. What are alternative ways to rotate the samples? Since catch-up saccades are followed by pursuit, we can use the direction of the immediately following smooth eye movement to rotate and align the samples. Unfortunately, SS events are mostly followed by fixations which do not have any direction. We can nevertheless compare our alignment algorithm with the gaze-based alignment by letting SVM classify CS1 against CS2, with each of the algorithms. The classification performance was poor for both sample alignment algorithms (0-3% over chance). Finally, one may be concerned that we made a mistake during the complex analysis. First, we note that in our experience in working with SVM, it is actually harder to produce poor performance. The SVM will exploit any bug during the extraction and filtering of the samples that differently affects ES and CS1 event. Second, we performed a CS1 vs. CS2 classification experiment where we aligned the samples with respect to the saccade direction. CS1 starts at rather random location and ends at the location of the tracked agents. CS2 starts and ends at the location of the tracked agents. Hence the CS2 direction is aligned with the agents' motion direction while the direction of CS1 is not aligned. The SVM exploited the difference in alignment and performed 4-8 % above the chance level. Thus, our implementation of the classification algorithm works, it just fails at the ES vs. CS1 classification

2 NON-PARAMETRIC REGRESSION ON GAZE COORDINATES

Why do we use coordinate representation instead of pixel representation? A pixel representation of a sample consists of $PxPxT$ values, where P is the number of pixels and T is the number of frames. In contrast, a sample in the coordinates consists of $2xAxT$ values, where we represent the position on the horizontal and vertical axis for each of A agents. A is mostly two, but there are pursuit episodes where subjects pursue one or more than two agents. To create template movies with high resolution P and T need to be large. As a consequence the pixel representation is computationally much more intensive.

The details of the analysis of pursuit templates are as follows: Smooth movement episodes are located between two consecutive catch-up saccades. The end of the preceding saccade marks the start of a pursuit episode and the episode ends with the onset of the next saccade. We performed multiple analyses with start, middle, and end of the pursuit episode as time lock. The positional lock varies across frames. It is given by the gaze position at the corresponding frame. The angle used for sample rotation and alignment also varies across frames. It is given by the direction of gaze movement between two consecutive frames. We obtain the value of the average template image I at position x and y and time t as $I(x,y,t) = \frac{1}{N}\sum_n^N w_n([x - x_{n,t}, y - y_{n,t}])$ where N is the total number of agents over all samples and $w_n(\mathbf{h}) \sim \exp\left(-\frac{1}{2}\mathbf{h}^\mathbf{T}\Sigma^{-1}\mathbf{h}\right)$ is the Gaussian function. Recall that we used Gaussian filter to smooth the pixel-based samples when we analyzed saccades. The Gaussian kernel does a similar job. Indeed, we use the same values for the standard deviation of the filter (Σ) as we did in the pixel-based analyses. In order to further improve the speed of the algorithm, we do not smooth over the time dimension.
